# MEK inhibition prevents CAR-T cell exhaustion and differentiation via downregulation of c-Fos and JunB

**DOI:** 10.1038/s41392-024-01986-y

**Published:** 2024-10-22

**Authors:** Xiujian Wang, Xiao Tao, Pengjie Chen, Penglei Jiang, Wenxiao Li, Hefeng Chang, Cong Wei, Xinyi Lai, Hao Zhang, Yihan Pan, Lijuan Ding, Zuyu Liang, Jiazhen Cui, Mi Shao, Xinyi Teng, Tianning Gu, Jieping Wei, Delin Kong, Xiaohui Si, Yingli Han, Huarui Fu, Yu Lin, Jian Yu, Xia Li, Dongrui Wang, Yongxian Hu, Pengxu Qian, He Huang

**Affiliations:** 1 Bone Marrow Transplantation Center, The First Affiliated Hospital, Zhejiang University School of Medicine, Hangzhou, 310003 China; 2 Liangzhu Laboratory, Zhejiang University Medical Center, 1369 West Wenyi Road, Hangzhou, 311121 China; 3 Institute of Hematology, Zhejiang University, Hangzhou, 310003 China; 4 Zhejiang Province Engineering Laboratory for Stem Cell and Immunity Therapy, Hangzhou, 310003 China; 5 Center of Stem Cell and Regenerative Medicine, Zhejiang University School of Medicine, Hangzhou, 310058 China; 6 Department of Hematology, The Third Affiliated Hospital of Wenzhou Medical University, Wenzhou, 325200 China

**Keywords:** Immunotherapy, Cancer therapy

## Abstract

Clinical evidence supports the notion that T cell exhaustion and terminal differentiation pose challenges to the persistence and effectiveness of chimeric antigen receptor-T (CAR-T) cells. MEK1/2 inhibitors (MEKIs), widely used in cancer treatment due to their ability to inhibit aberrant MAPK signaling, have shown potential synergistic effects when combined with immunotherapy. However, the impact and mechanisms of MEKIs on CAR-T cells remain uncertain and controversial. To address this, we conducted a comprehensive investigation to determine whether MEKIs enhance or impair the efficacy of CAR-T cells. Our findings revealed that MEKIs attenuated CAR-T cell exhaustion and terminal differentiation induced by tonic signaling and antigen stimulation, thereby improving CAR-T cell efficacy against hematological and solid tumors. Remarkably, these effects were independent of the specific scFvs and costimulatory domains utilized in CARs. Mechanistically, analysis of bulk and single-cell transcriptional profiles demonstrates that the effect of MEK inhibition was related to diminish anabolic metabolism and downregulation of c-Fos and JunB. Additionally, the overexpression of c-Fos or JunB in CAR-T cells counteracted the effects of MEK inhibition. Furthermore, our Cut-and-Tag assay revealed that MEK inhibition downregulated the JunB-driven gene profiles associated with exhaustion, differentiation, anergy, glycolysis, and apoptosis. In summary, our research unveil the critical role of the MAPK-c-Fos-JunB axis in driving CAR-T cell exhaustion and terminal differentiation. These mechanistic insights significantly broaden the potential application of MEKIs to enhance the effectiveness of CAR-T therapy.

## Introduction

Oncogenic mutation targeted therapy has shown responses in tumors harboring mutated oncogenes such as mutant BRAF.^1^ However, targeted therapy is generally not curative because secondary mutations and transcriptional alterations often confer resistance to the primary therapy.^2^ Chimeric antigen receptors (CARs) are synthetic receptors targeting tumor antigens.^3^ CAR-T therapy has shown potent efficacy in relapsed or refractory B-cell malignancies,^3^ even those resistant to targeted therapy.^4^ Nevertheless, the limited persistence of CAR-T cells due to T cell exhaustion and terminal differentiation has restricted post-CAR-T survival of patients in both B-cell malignancies and solid tumors.^3,5^

Chronic CAR signaling is one of the intrinsic causes of CAR-T cell exhaustion and terminal differentiation.^6,7^ According to whether the initiation of CAR signaling needs antigen binding, CAR signaling can be categorized into low-level basal tonic signaling (independent of antigen stimulation),^6,8,9^ and antigen-dependent high-level signaling.^7,9^ Tonic signaling results from the self-aggregation of CAR molecules independent of antigen stimulation.^6,10^ Thus, its downstream signaling cascade molecules are the same as those activated by CAR clustering due to antigen binding. The primary differences between tonic signaling and antigen-dependent signaling are the trigger (with or without antigen stimulation) and the signaling intensity (low versus high). Considering that many targets of targeted agents are also crucial signaling molecules in CAR-T cells, combining CAR-T and targeted therapy may be a promising way to prolong the persistence of CAR-T cells and overcome the shortcomings of respective monotherapy. Unfortunately, the inhibition of these signaling molecules might also impair the effector function of CAR-T cells.^11^ Thus, studies are needed to evaluate the effect of every targeted agent on CAR-T cells to seek the optimal combining regimens to treat tumors with specific mutations.

Genetic mutations in mitogen-activated protein kinase (MAPK) signaling pathway are among the most prevalent in human cancers,^2^ which leads to aberrant activation of MAPK signaling. The canonical cascade of MAPK signaling pathway is RAS-RAF-MEK1/2-ERK1/2 cascade.^2^ MEK1/2 inhibitors (MEKIs) have been used to treat tumors carrying RAF or RAS mutations.^1^ Intriguingly, emerging studies have shown that the antitumor effect of MEKIs depended not only on directly inhibiting tumor MAPK signaling but also on modulating immunity.^12^ These effects of MEKIs are partly mediated through regulating T cell differentiation and tumor microenvironment and preventing T cell death.^12,13^ These mechanistic insights promote a clinical trial evaluating the combination of PD-1, BRAF, and MEK inhibition in solid tumors, which yielded favorable results.^14^ However, the effects of MEKIs on T cell exhaustion and the molecular mechanisms MEKIs harness to regulate T cells are not fully understood. Most studies about the effects of MEKIs on T cells were conducted on murine T cells. The impacts of MEKIs on human T cells, especially CAR-T cells, are poorly understood and remain controversial.^15^ On the one hand, two groups showed that MEKIs could impair CAR-T cells’ effector function in vitro.^16,17^ On the other hand, another group reported that MEKIs could enhance the efficacy of GD2 CAR-T cells manufactured by a piggyBac transposon (PB)-based protocol in mouse models.^18^ Nevertheless, the mechanism for the combined effect is unclear. Whether MEKIs benefit CAR-T cells manufactured by mainstream lentivirus-based protocols remains unknown. Thus, studies are urgently needed to evaluate the impacts of MEKIs on CAR-T cells and elucidate the mechanisms.^15^

Given that the CAR signaling pathway is similar to the TCR signaling pathway, both of which involve the activation of MAPK signaling pathway^19^; we hypothesize that MEKIs might mitigate CAR-T cells’ exhaustion and terminal differentiation by lessening the redundant CAR signaling. Nowadays, FDA has approved several MEKIs, including trametinib, cobimetinib, and binimetinib.^1,20^ Among them, trametinib is the most potent, with the lowest IC50 and administration dose and the longest in-vivo half-life.^1,20^ Its specificity for MEK1/2 has been confirmed against a panel of more than 180 kinases, including B-Raf, C-Raf, and the closest kinase homolog MEK5,^21^ and its peak concentration in blood is 36 nM.^1^ We demonstrate that MEKIs, especially trametinib, can reduce CAR-T cells’ activation, exhaustion, apoptosis, and terminal differentiation in tonic signaling and antigen stimulation contexts. Incorporating MEKIs into CAR-T cell manufacturing yields cells with more central memory, diminished exhaustion, and enhanced in-vivo efficacy. Moreover, the in-vivo administration of MEKIs and CAR-T cells produces better antitumor effects than respective monotherapy. The role of MEKIs on CAR-T cells is associated with transcriptional reprogramming characterized by the upregulation of memory-related genes and downregulation of activation-, exhaustion-, anabolic metabolism-related, and pro-apoptotic genes. Further, the downregulation of AP-1 transcription factors c-Fos and JunB play a central role in driving the transcriptional reprogramming initiated by MEKIs. Overexpression of c-Fos or JunB in CAR-T cells abrogates the effects of MEK inhibition. In summary, we propose a novel strategy to augment the antitumor efficacy of CAR-T therapy and MEK inhibitors by combining them and highlight the role of c-Fos and JunB in driving CAR-T cells’ exhaustion and terminal differentiation.

## Results

### MEKIs restrain CAR-T cell exhaustion and differentiation elicited by antigen-independent tonic signaling

**Fig. 1:**
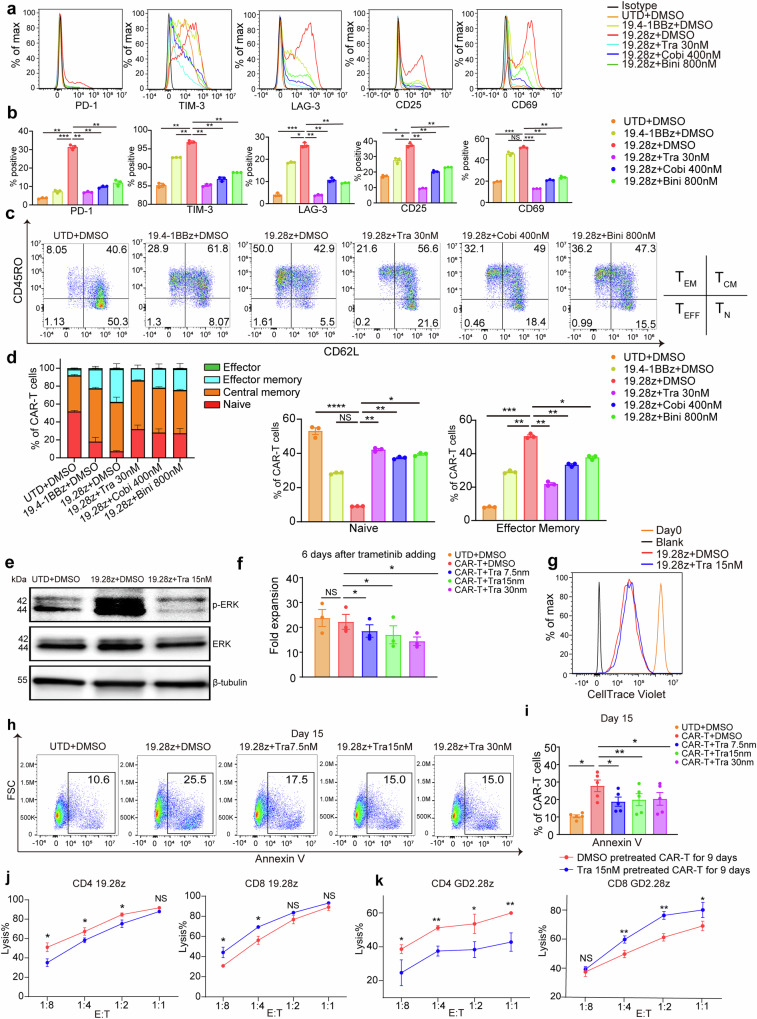
MEK inhibition restrains the exhaustion and differentiation of CAR-T cells elicited by antigen-independent tonic signaling. **a**, **b** Flow cytometric analysis of exhaustion (PD-1, TIM-3, and LAG-3) and activation markers (CD25 and CD69) of CAR-T cells on day 15 of ex-vivo culture. One representative donor’s histograms (**a**) are shown. Bar graphs (**b**) are pooled from 3 donors. **c**, **d** Differentiation state of CAR-T cells on day 15 of ex-vivo culture. One representative donor’s pseudocolor plots are shown (**c**). CD62L and CD45RO define the differentiation state of CAR-T cells as the following combinations: naive T cells (T_N_, CD62L + CD45RO-), central memory T cells (T_CM_, CD62L + CD45RO + ), effector memory T cells (T_EM_, CD62L-CD45RO + ) and effector T cells (T_EFF_, CD62L-CD45RO-). Bar graphs (**d**) are pooled from 3 donors. **e** WB evaluates pERK versus total ERK of 19.28z CAR-T cells on day 15 of ex-vivo culture. Representative of 3 donors. **f** The bar graphs show the expansion fold of 19.28z CAR-T cells. Data are pooled from 3 donors. **g** Representative histogram of CellTrace Violet showing the cell proliferation state of 19.28z CAR-T cells after a six-day treatment with trametinib. *N* = 2 donors. **h**, **i** Quantification of apoptosis of 19.28z CAR T cells on day 15 of ex-vivo culture. One representative donor’s pseudocolor plots (**h**) are shown. Bar graphs (**i**) are pooled from 5 donors. **j**, **k** Cytotoxicity of CD4 and CD8 19.28z CAR-T cells (**j**), and CD4 and CD8 GD2.28z CAR-T cells (**k**) pre-treated with trametinib for 9 days. The assay was conducted in culture media without trametinib. Error bars represent means ± SD of triplicate wells. A representative donor from three donors. Error bars are means ± SEM and statistical test was paired one-way ANOVA with Dunnett’s multiple comparison test except (**j**, **k**). For (**j**, **k**), an unpaired student’s *t*-test was used. *P < 0.05, **P < 0.01, ***P < 0.001, ****P < 0.0001, NS not significant, Tra trametinib, Cobi cobimetinib, Bini binimetinib, UTD untransduced T

The study utilized GD2.28z, CD19.28z (19.28z), and CD19.4-1BBz (19.4-1BBz) CARs (Supplementary Fig. 1a). CAR tonic signaling gradually decreased across these 3 CARs, resulting from the difference in their scFvs and costimulation domains.^6,10^ Untransduced T (UTD) and 19.4-1BBz CAR-T cells expressed fewer inhibitory receptors (PD-1, TIM-3, and LAG-3) and activation markers (CD25 and CD69) and contained fewer effector memory CAR-T cells compared to 19.28z CAR-T cells (Fig. 1a–d). Using UTD cells and 19.4-1BBz CAR-T cells as controls, we first added trametinib, cobimetinib, and binimetinib, respectively, to 19.28z CAR-T cell culture at concentrations approaching their peak blood concentration for 9 days.^1,20^ All 3 MEKIs increased naive, reduced effector memory, and inhibited the expression of inhibitory receptors and activation markers (Fig. 1a–d). Among them, trametinib was the most potent because it could achieve the comparable effect of cobimetinib and binimetinib at the lowest concentration (Fig. 1a–d). Thus, we chose trametinib for further research. Compared to 7.5 nM and 30 nM, at 15 nM, trametinib demonstrated optimal efficacy by mildly inhibiting CAR-T cell expansion while effectively suppressing the activation, exhaustion, apoptosis, differentiation, CD8 decline, and ERK phosphorylation of CAR-T cells (Fig. 1e–i and Supplementary Fig. 1b–g). The above impacts of trametinib were more significant as the treatment time increased (Supplementary Fig. 1b–g). The roles of trametinib in reducing expression of exhaustion and activation markers and differentiation of CD4 and CD8 single-positive CAR-T cells were the same as its roles on bulk CAR-T cells (Supplementary Fig. 2a–d). A 9-day pretreatment with trametinib significantly reduced the release of TNF-α, IFN-γ, IL2, GzmB, CCL3, and CXCL8 by CD4 19.28z cells (Supplementary Fig. 2e). In contrast, the same pretreatment slightly inhibited CD8 19.28z CAR-T cells from releasing TNF-α, GzmB, CCL3, and CXCL8 while enhancing their release of IL2 and IFN-γ (Supplementary Fig. 2f). Consistently, a 9-day pretreatment with trametinib impaired the cytotoxicity of CD4 19.28z CAR-T cells but enhanced the cytotoxicity of CD8 19.28z CAR-T cells, while having no impact on the cytotoxicity of bulk 19.28z CAR-T cells (Fig. 1j and Supplementary Fig. 1h).

To confirm whether the effects of MEKIs depend on the specific scFv and costimulation domain, we treated 19.4-1BBz and exhaustion-susceptible GD2.28z CAR-T cells^6,10,22^ with trametinib. Consistently, trametinib prevented them from activation, exhaustion, apoptosis, and differentiation (Supplementary Fig. 3a–j). Interestingly, trametinib mildly inhibited cell proliferation in the less-exhausted 19.28z and 19.4-1BBz CAR-T cells while slightly promoting proliferation in the more exhaustion-prone GD2.28z CAR-T cells^6,10,22^ (Fig. 1f and Supplementary Fig. 3l). Consistently, cell trace violet showed that trametinib had a minimal effect on the cell division of these three types of CAR-T cells (Fig. 1g and Supplementary Fig. 3k). Both methods confirmed that trametinib had a mild effect on CAR-T cell proliferation. Likely due to the higher exhaustion level in GD2.28z CAR-T cells, trametinib’s anti-apoptotic effect resulted in a slight increase in their cell quantity during manufacturing.

In line with the effect on 19.28z CAR-T cells, a 9-day trametinib pretreatment decreased the effector function of CD4 GD2.28z CAR-T cells while boosting the effector function of CD8 GD2.28z CAR-T cells (Fig. 1k and Supplementary Fig. 2g, h). Notably, trametinib had a more pronounced positive impact on the effector function of CD8 GD2.28z CAR-T cells compared to 19.28z CAR-T cells (Fig. 1j, k and Supplementary Fig. 2f–h) and even promoted the cytotoxicity of bulk GD2.28z CAR-T cells (Supplementary Fig. 1i).

**Fig. 2:**
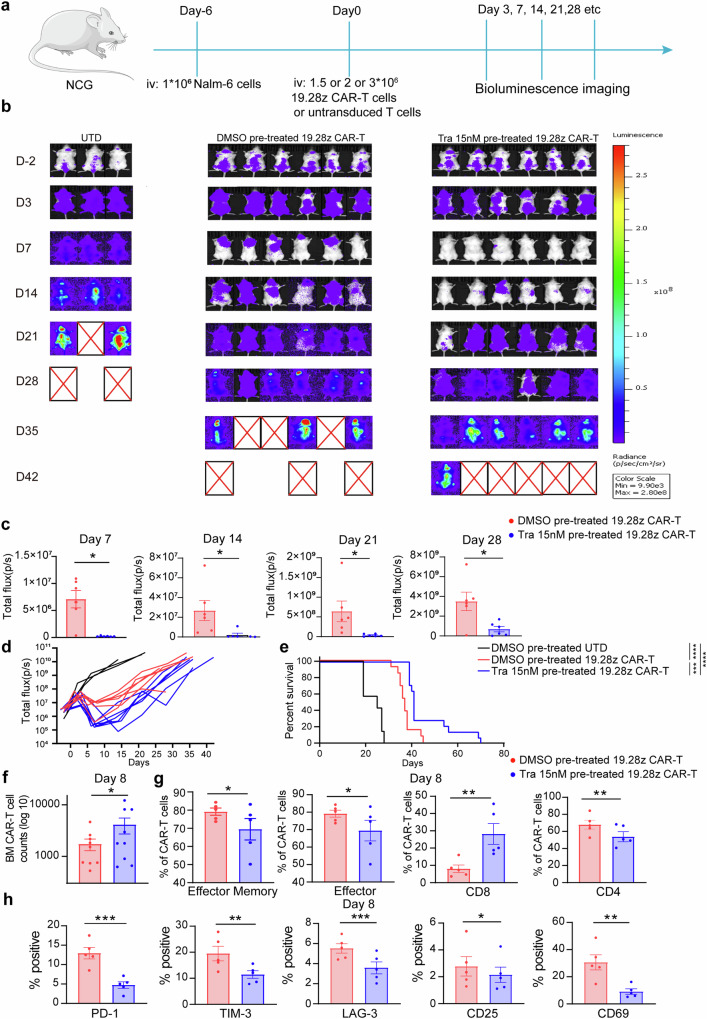
Trametinib pretreatment during ex-vivo manufacturing enhances the in-vivo efficacy of 19.28z CAR-T cells. **a** 1.5 or 2 or 3 × 10^6^ 19.28z CAR-T cells manufactured in the presence of trametinib 15 nM or DMSO for 9 days were infused to NCG mice intravenously (IV) 6 days after engraftment of 1 × 10^6^ Nalm-6-GL leukemia cells in 3 independent experiments. **b**–**d** Tumor growth was monitored by bioluminescent imaging. One representative experiment is shown, and 1.5 * 10^6^ CAR-T cells were administrated (UTD cells: *n* = 3; DMSO pre-treated CAR-T cells: *n* = 6; trametinib 15 nM pre-treated CAR-T cells: *n* = 6). Each dot in (**c**) and each curve in (**d**) represents one mouse. D, day. **e** Kaplan–Meier analysis of survival of mice. Data are pooled from 3 independent experiments (UTD cells: *n* = 7; DMSO pre-treated CAR-T cells: *n* = 13; trametinib 15 nM pre-treated CAR-T cells: *n* = 14). **f**–**h** The number (**f**), subset composition (**g**), and positive rate of exhaustion and activation markers (**h**) of CAR-T cells in the bone marrow 8 days after CAR-T infusion. Data in (**f**) are pooled from 2 independent experiments, with 1.5 * 10^6^ and 2 * 10^6^ CAR-T cells administered in each experiment, respectively. Data in (**g**, **h**) are from 1 representative experiment, with 1.5 * 10^6^ CAR-T cells administered in (**g**) and 2 * 10^6^ CAR-T cells administered in (**h**). Each dot represents one mouse. *N* = 5 or 9. Error bars are means ± SEM. Statistical tests were paired two-tailed Wilcoxon test (**c**) and paired two-tailed student’s *t*-test (**f**–**h**). Survival curves were compared using a log-rank Mantel-Cox test. *P < 0.05, **P < 0.01, ***P < 0.001, ****P < 0.0001. D day, Tra trametinib, UTD untransduced T

We further investigated the in-vivo efficacy of trametinib pretreated CAR-T cells using a leukemia xenograft model in NCG mice (Fig. 2a). Trametinib pretreated 19.28z CAR-T cells demonstrated superior anti-leukemia activity compared to DMSO pretreated counterparts. That was evidenced by lower leukemia burden, longer mice survival, improved CD8 CAR-T expansion, increased CAR-T infiltration in bone marrow, more effector memory CAR-T cells, fewer effector CAR-T cells, and diminished expression of exhaustion and activation markers (Fig. 2b–h). We obtained similar results in xenograft leukemia model treated with 19.4-1BBz CAR-T cells (Supplementary Fig. 4a–h).

Overall, regardless of scFv and costimulation domain, trametinib treatment during ex-vivo manufacturing prevented CAR-T cells from overactivation, exhaustion, apoptosis, differentiation, and CD8 decline driven by tonic signaling, thus enhancing CAR-T cells’ in-vivo potency.

### MEKIs modify CAR-T cell transcriptome during ex-vivo manufacturing

To explore the molecular mechanism underlying the enhanced persistence of trametinib-treated CAR-T cells, we performed transcriptional profiling of DMSO-treated UTD cells and DMSO or trametinib-treated 19.28z CAR-T cells (without antigen or tumor cell stimulation). 2128 DEGs among the three groups were identified, with 544 DEGs shared between CAR-T + DMSO vs. UTD + DMSO and CAR-T + DMSO vs. CAR-T + trametinib (Supplementary Fig. 5a).

**Fig. 3:**
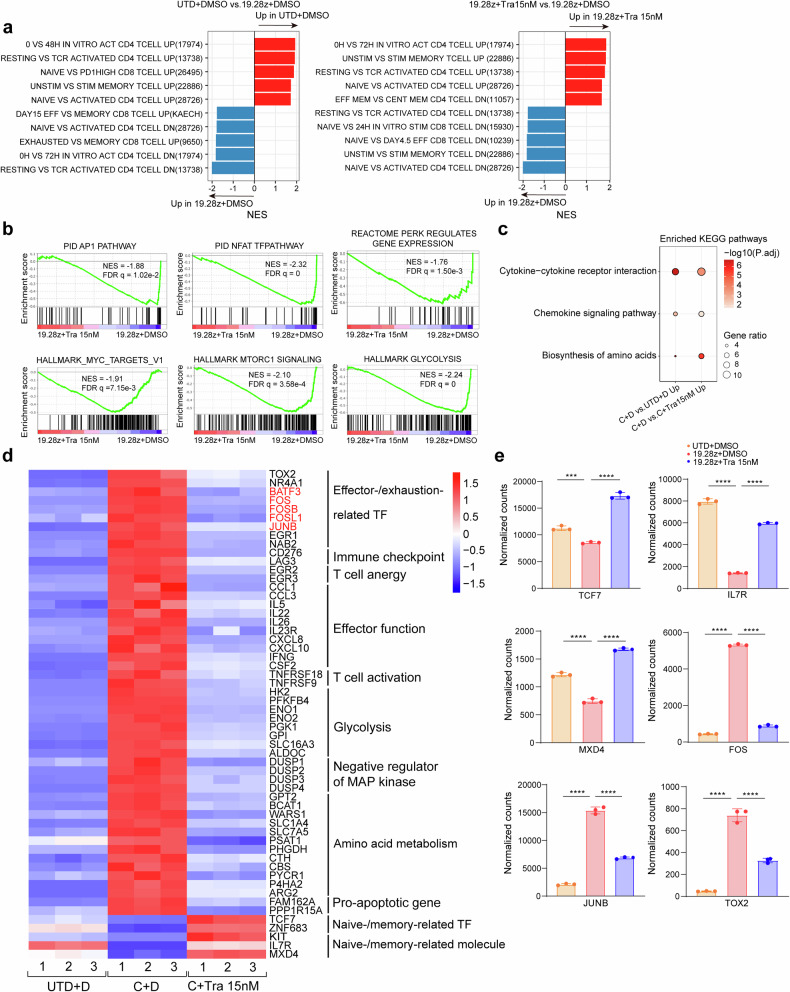
MEK inhibition modifies the gene-expression profile of CAR-T cells during ex-vivo manufacturing. **a** Normalized enrichment score (NES) of significantly up- or downregulated gene sets in UTD + DMSO versus 19.28z CAR-T + DMSO (left panel) and 19.28z CAR-T + trametinib 15 nM versus 19.28z CAR-T + DMSO (right panel) as determined by GSEA using the MSigDB C7 gene sets. For all pathways, the false discovery rate (FDR) < 0.05. **b** Representative GSEA enrichment plot. **c** KEGG pathway enrichment analysis of differentially expressed genes (DEGs) among samples. **d** Heat map demonstrating the expression profiles of selected DEGs (FDR < 0.05) among the three groups. The AP-1 TFs are denoted in red. **e** Normalized RNA-seq counts of selected genes. Error bars are means ± SEM. Statistical test was unpaired one-way ANOVA with Dunnett’s multiple comparison test. For all analyses, *n* = 3 per group. ***P < 0.001, ****P < 0.0001. UTD untransduced T. C 19.28z CAR-T, D DMSO, Tra trametinib,TF transcription factor

GSEA showed DMSO-treated group downregulated naive-/memory-related genes and upregulated Myc- and pERK-regulated genes, and genes involved in T cell activation/effector/exhaustion, AP-1/NFAT/mTORC1 pathway, cytokine signaling and glycolysis/amino acid metabolism, compared to UTD and trametinib-treated group (Fig. 3a, b and Supplementary Fig. 5b, c). Consistently, KEGG pathways enriched in the DMSO-treated group relative to UTD and the trametinib-treated group included cytokine−cytokine receptor interaction, chemokine signaling pathway, and amino acids biosynthesis (Fig. 3c). The results of GSEA and KEGG were in accordance with the phenotypic change caused by trametinib during CAR-T manufacturing.

Next, we examined specific gene alterations during the process. Naive-/memory-related TFs (*TCF7*, etc.)^23^ and *MXD4*, a suppressor of *MYC*,^24^ were upregulated with *KIT* and *IL7R* acquisition, whereas effector/exhaustion-related TFs (*EGR1*, *TOX2 and NR4A1*, etc.),^25,26^ AP-1 TFs (*FOS* and *JUNB*, etc.)^22^ and anergy-related TFs (*EGR2*, etc.)^27^ were downregulated with immune checkpoints (*LAG3*, etc.), effector molecules (*CCL1*, etc.) and pro-apoptotic genes (*FAM162A*, etc.) reduction in UTD and trametinib-treated group relative to DMSO-treated group (Fig. 3d, e, Supplementary Fig. 5d and Supplementary Table 1). Additionally, trametinib downregulated MAP kinase suppressors (*DUSP1*, etc.) and enzymes implicated in glycolysis (*HK2*, etc.) and amino acid metabolism (*GPT2*, etc.). The former probably served as a feedback mechanism,^28^ while the latter implied a more quiescent metabolic state in CAR-T cells following MEK inhibition (Fig. 3d, e, supplementary Fig. 5d and Supplementary Table 1).

Finally, we investigated how trametinib affects the transcriptional profile of 19.4-1BBz CAR-T cells during their manufacturing process. Trametinib treatment led to 126 shared DEGs in 19.4-1BBz and 19.28z CAR-T cells. They shared downregulated genes, including exhaustion-related TFs (*EGR1*, *TOX2 and NR4A1*),^25,26^ AP-1 TFs (*FOS* and *FOSL1*),^22^ anergy-related TFs (*EGR2* and EGR3),^27^ an immune checkpoint (*CD276*), effector molecules (*CCL1*, *CCL3* and *CXCL8*, etc), a T cell activation marker (*TNFRSF18*) and negative regulators of MAP kinase (*DUSP2, DUSP4, SPRED2*) (Supplementary Fig. 5e). Of note, the shared upregulated genes included the stemness-related molecule *KIT* (Supplementary Fig. 5e)

### MEKIs limit antigen-induced CAR-T cell exhaustion and differentiation

**Fig. 4:**
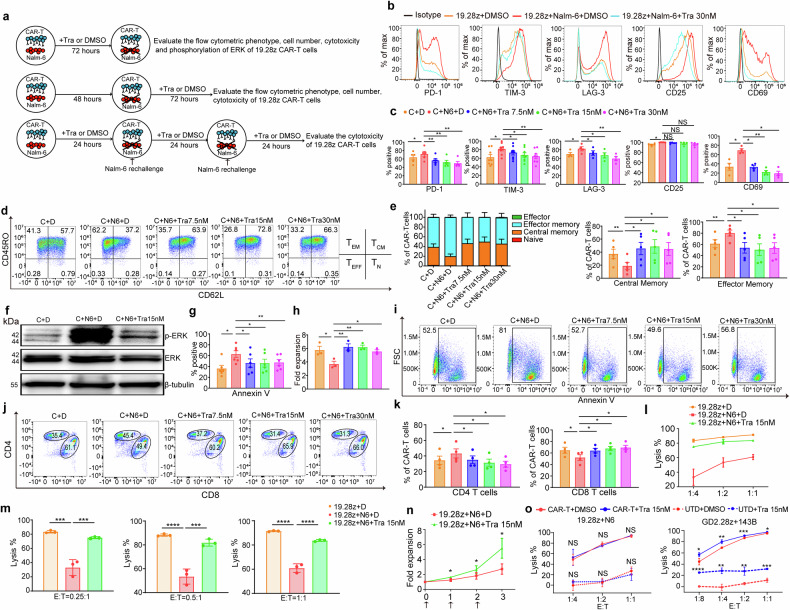
MEK inhibition limits the exhaustion and differentiation of CAR-T cells triggered by target antigen. **a** Experimental design for target antigen stimulation. **b**, **c** Flow cytometric analysis of exhaustion and activation markers. The histograms (**b**) of one representative donor are shown. Bar graphs (**c**) are pooled from 4 to 8 donors. **d**, **e** Differentiation state of 19.28z CAR-T cells. Pseudocolor plots (**d**) of one representative donor are shown. Bar graphs (**e**) are pooled from 5 donors. (**f**) WB evaluates pERK versus total ERK. Representative of 3 donors. **g**, **i** Quantification of apoptosis of 19.28z CAR T cells. The bar graphs (**g**) are pooled from 6 donors and the pseudocolor plots (**i**) of one representative donor are shown. (**h**) The bar graphs show the expansion fold of 19.28z CAR-T cells. Data are pooled from 3 donors. **j**, **k** CD8 and CD4 composition in 19.28z CAR-T cells. Pseudocolor plots (**j**) of one representative donor are shown. Bar graphs (**k**) are pooled from 4 donors. **l**, **m** Cytotoxicity of 19.28z CAR-T cells cocultured with Nalm-6-GL cells for 18 h in the culture medium free of trametinib or DMSO. Error bars represent means ± SD of triplicate wells. A representative donor from four donors. **n** Proliferation curve of 19.28z CAR-T cells. Arrows denoted the time points when Nalm-6 cells were added. Data are pooled from 5 donors. **o** Cytotoxicity of 19.28z CAR-T against Nalm-6-GL cells (left panel) and GD2.28z CAR-T against 143B-GL cells (right panel) in the culture medium with trametinib or DMSO. Error bars represent means ± SD of triplicate wells. (19.28z: *n* = 4 donors; GD2.28z: *n* = 3 donors). The data in (**b**–**k**) were produced using CAR-T cells treated by protocols indicated in the upper panel of Fig. 4a. Error bars are means ± SEM unless indicated otherwise. Statistical tests were paired (**c**, **e**, **g**, **h**, **k**) or unpaired (**m**) one-way ANOVA with Dunnett’s multiple comparison test, and paired (**n**) or unpaired (**o**) students’ *t* test. Tests were two-tailed. *P < 0.05, **P < 0.01, ***P < 0.001, ****P < 0.0001.NS not significant. C 19.28z CAR-T, D DMSO, Tra trametinib, N6 Nalm-6, UTD untransduced T

To evaluate trametinib’s protection against antigen-induced CAR-T cell exhaustion and differentiation, we conducted three in-vitro assays with 19.28z CAR-T cells (Fig. 4a and “Methods”). Whether added at the beginning of coculture or after Nalm-6 cells eradication, trametinib can reduce exhaustion, activation, and ERK phosphorylation, prevent differentiation, apoptosis, and CD8 decline, and promote proliferation of CAR-T cells (Fig. 4b–k and Supplementary Fig. 6a–i). CD25 MFI was lowered, but the change in CD25 positive rate pooled from 5 donors was statistically insignificant (Fig. 4b, c and Supplementary Fig. 6a, b). Again, 15 nM was the optimal concentration of trametinib for its relatively maximum effects in promoting CAR-T expansion and ameliorating exhaustion and differentiation (Fig. 4b–e, g–i and Supplementary Fig. 6a–g).

Probably due to insufficient one-time antigen stimulation, trametinib didn’t enhance CAR-T cells’ cytotoxicity after it was washed out (Supplementary Fig. 6j, k). Thus, we designed the repetitive antigen stimulation assay (Fig. 4a, the lowest panel). Expectedly, trametinib reduced CAR-T cells’ functional exhaustion and growth arrest under this situation (Fig. 4l–n).

Previous cytotoxicity assays used the culture media without trametinib. Given trametinib’s impact on reducing CAR-T cell activation, we investigated its influence on CAR-T cell effector function. 19.28z and GD2.28z CAR-T cells were cocultured with Nalm-6-GL and 143B-GL cells, respectively, in culture media containing DMSO or trametinib. Trametinib reduced the release of TNF-α, IFN-γ, IL2, GzmB, CCL3, and CXCL8 in both 19.28z and GD2.28z CAR-T cells, regardless of the CD4 and CD8 subsets (Supplementary Fig. 6l–o). However, trametinib didn’t impair 19.28z CAR-T cells’ cytotoxicity against Nalm-6 cells (Fig. 4o). Furthermore, trametinib showed direct killing against 143B cells, and when combined with GD2.28z CAR-T cells, trametinib and GD2.28z CAR-T cells showed synergistic killing in vitro (Fig. 4o).

**Fig. 5:**
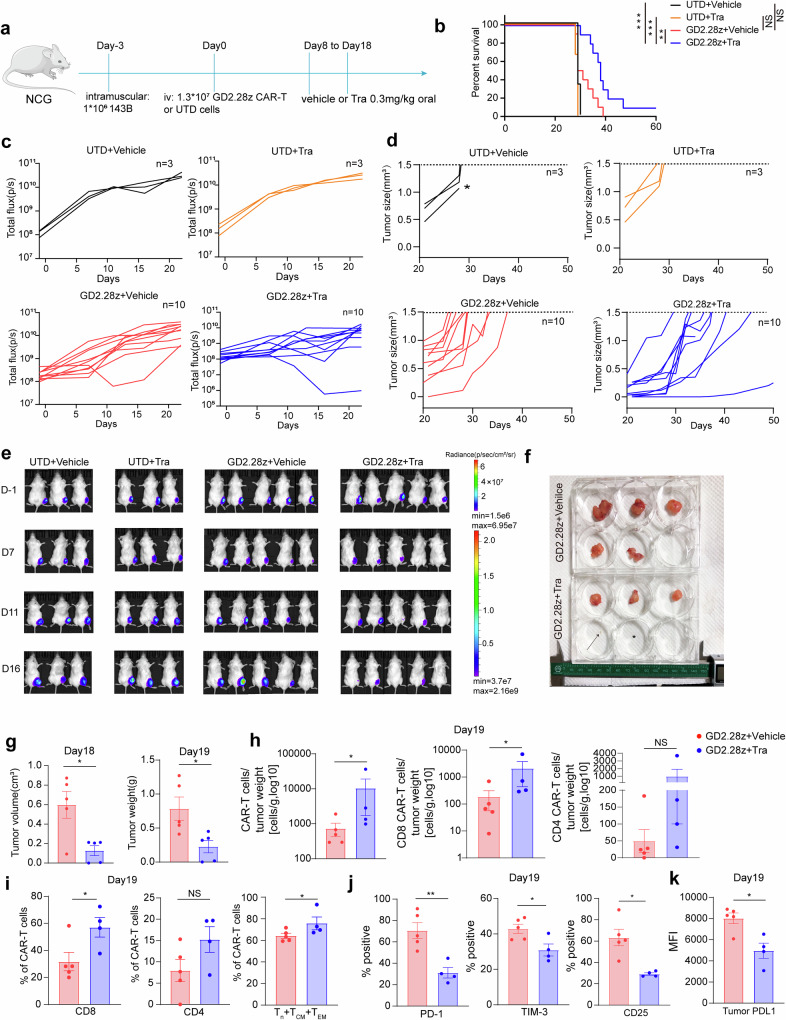
In-vivo administration of trametinib enhances the antitumor activities of CAR-T cells by offsetting CAR-T cells exhaustion and differentiation. **a** Mice treated with GD2.28z CAR-T cells or UTD cells were dosed with trametinib or vehicle once daily. **b** Kaplan–Meier analysis of survival of mice. Data are pooled from two independent experiments (UTD + vehicle: *n* = 3; UTD + trametinib: *n* = 3; GD2.28z + vehicle: *n* = 10; GD2.28z + trametinib: *n* = 10). **c**, **e** Tumor growth was monitored by bioluminescent imaging. Each curve in (**c**) represents one mouse. D day. **d** Growth curve of tumor size. Each line represents one mouse. The dotted lines indicate the endpoint where tumor volume reached 1.5 cm^3^, and the asterisks indicate mice died before reaching the endpoint. **f** Picture of tumors on day 19 after CAR-T infusion. The arrow indicates a tiny tumor, and the asterisk indicates a missing macroscopic tumor in one of the mice in GD2.28z + trametinib group. *N* = 5 mice per group. One of two independent experiments is shown. **g** Bar plots of tumor volume and tumor weight of mice from (**f**). *N* = 5 mice per group. **h** Tumor-infiltrating total GD2.28z, CD8 GD2.28z, and CD4 GD2.28z CAR-T cell counts normalized to tumor weight of mice from (**g**). GD2.28z + vehicle: *n* = 5. GD2.28z + trametinib: *n* = 4 for one mouse in GD2.28z CAR-T + trametinib group didn’t have a macroscopic tumor. **i** The percentage of CD8 and CD4, along with the total percentage of naïve plus central memory plus effector memory in tumor-infiltrating GD2.28z CAR-T cells of mice from (**g**). **j** The positive rate of PD-1, TIM-3, and CD25 in tumor-infiltrating GD2.28z CAR-T cells of mice from (**g**). **k** The MFI of PDL1 in tumor cells of mice from (**g**). Error bars are means ± SEM unless indicated otherwise. Statistical tests were unpaired (**g**, **i**, **j**, **k**) students’ *t* test, Mann–Whitney test (**h**), and log-rank Mantel-Cox test (**b**). All tests were two-tailed. *P < 0.05, **P < 0.01, ***P < 0.001, NS not significant, Tra trametinib, UTD untransduced T, N naive, CM central memory, EM effector memory

We next investigated if trametinib in-vivo administration prevented CAR-T cells from exhaustion and differentiation. To exclude MEKIs’ potential suppression on 143B cells, we injected 143B-bearing mice with UTD cells or GD2.28z CAR-T cells and orally administrated mice with trametinib or vehicle (Fig. 5a). Likely due to the differences between in-vitro and in-vivo circumstances, unlike the in-vitro killing assay, after tumor engraftment, administrating trametinib alone following our protocol from day 8 to 18 didn’t mitigate tumor progression (Fig. 5b–e). Before day 15, CAR-T cells alone only slowed tumor growth in 1 out of 10 mice and didn’t significantly increase mice survival (Fig. 5b, c). In contrast, the combination of trametinib and CAR-T cells significantly delayed tumor progression and extended mice survival, with one mouse having no visible tumor at euthanasia (Fig. 5b–g). Tumor analysis showed an increase in total and CD8 CAR-T cells, as well as the sum of naïve plus central memory plus effector memory CAR-T cells (Fig. 5h, i), with lower PD-1, TIM-3, and CD25 expression (Fig. 5j). Trametinib reduced PDL1 expression in tumor cells, consistent with prior research showing MEK-mediated RAS signaling increased PDL1 expression^29^ (Fig. 5k). To investigate the optimal combination protocols of MEKIs and CAR-T cells, we further compared pulsatile vs continuous MEK inhibition on CAR-T cells using CAR-T cells from another donor (Supplementary Fig. 7a). Again, trametinib alone didn’t mitigate tumor progression (Supplementary Fig. 7b–f). GD2.28z CAR-T cells from this donor slightly slowed tumor progression and minimally extended mice survival (Supplementary Fig. 7b–f). Conversely, continuous MEK inhibition on CAR-T cells notably delayed tumor progression and extended mice survival in all five mice (Supplementary Fig. 7b–f). Although there was no statistically significant difference in tumor burden and mice survival between continuous and pulsatile MEK inhibition, the pulsatile group exhibited trends of higher average tumor burden and shorter median survival (49.5 vs 53 days) compared to the continuous group, indicating continuous MEK inhibition is the optimal combination ways of MEKIs and CAR-T cells. Together, these data implied that under antigen stimulation, MEKIs reduced CAR-T cell exhaustion and differentiation both in vitro and in vivo.

### MEKIs alter antigen-stimulated CAR-T cells transcriptome to a naive-/memory-like state

We analyzed the transcriptome of Nalm-6-stimulated CAR-T cells treated with DMSO or trametinib and CAR-T cells without Nalm-6 stimulation (defined as “resting” below). After antigen stimulation, the transcriptomic differences among the three groups differed from the “resting state.” 5337 DEGs among the three groups were identified, with 1070 DEGs shared between CAR-T + Nalm-6 + DMSO vs. CAR-T + DMSO and CAR-T + Nalm-6 + DMSO vs. CAR-T + Nalm-6 + trametinib (Supplementary Fig. 8a), suggesting the alteration of MEKIs on CAR-T cell transcriptome is magnified under antigen exposure (Supplementary Figs. 5a, 8a). Of the 1576 DEGs in CAR-T + Nalm-6 + DMSO vs. CAR-T + Nalm-6 + trametinib, 270 overlapped with CAR-T + DMSO vs. CAR-T + trametinib (Supplementary Fig. 8b). Trametinib-treated groups shared downregulated genes, including exhaustion/anergy-related and AP-1 TFs (*FOS* and *JUNB*, etc.), effector molecules, glycolysis/amino acid metabolism-related enzymes, and pro-apoptotic genes. They also shared upregulated genes, including naive-/memory-related genes, *KIT*, and MXD4 (Supplementary Fig. 8b).

**Fig. 6:**
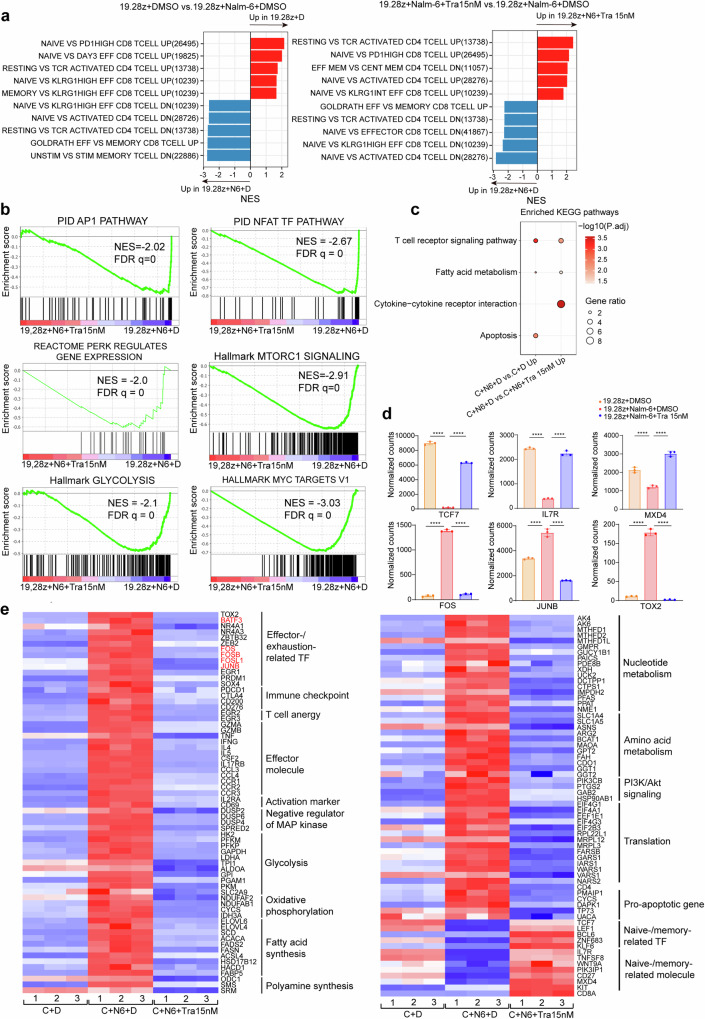
MEK inhibition alters the gene profile of antigen-stimulated CAR-T cells to a naive-/memory-like state. (**a**) NES of significantly up- or downregulated gene sets in 19.28z + DMSO versus 19.28z + Nalm-6 + DMSO (left panel) and 19.28z + Nalm-6 + trametinib 15 nM versus 19.28z + Nalm-6 + DMSO (right panel), as determined by GSEA using the MSigDB C7 gene, sets. For all pathways, the FDR *q* < 0.05. **b** Representative GSEA enrichment plot. **c** KEGG pathway enrichment analysis of DEGs among samples. **d** Normalized RNA-seq counts of selected genes. Statistical test was unpaired two-tailed one-way ANOVA with Dunnett’s multiple comparison test. Error bars are means ± SEM. **e** Heat map showing the expression profiles of selected DEGs (FDR < 0.05) among the three groups. The AP-1 TFs are denoted in red. For all analyses, *n* = 3 per group. C 19.28z CAR-T, N6 Nalm-6, D DMSO, Tra trametinib., TF transcription factor

Apart from the differential pathways found in the “resting state”, GSEA revealed Nalm-6-stimulated group upregulated several novel pathways, including oxidative phosphorylation, fatty acid/nucleotide/polyamine metabolism, and apoptosis compared to trametinib-treated and resting groups (Fig. 6a, b and Supplementary Fig. 8c, d). The newly-found KEGG pathways enriched in Nalm-6-stimulated group relative to trametinib-treated and resting groups included T cell receptor signaling pathway and fatty acid metabolism (Fig. 6c). Given the similarity between CAR signaling pathway and TCR signaling pathway,^19,30^ these results confirmed that under antigen stimulation, MEK inhibition not only suppresses genes associated with the CAR signaling pathway but also appears to attenuate the metabolic activities of CAR-T cells.

Besides the naïve/memory-related and exhaustion/effector/anergy/glycolysis/amino acid metabolism/apoptosis-related DEGs found in “resting state”, a suppressor of PI3K/Akt/mTOR pathway, *PIK3IP1*^31^ was upregulated, whereas genes participating in mitochondria oxidative phosphorylation (*NDUFAF2*, etc.), polyamine synthesis (*ODC1*, etc), nucleotide metabolism (*AK4*, etc.), and translation (*EIF4A1*, etc.) were downregulated in trametinib-treated and resting groups relative to DMSO-treated group, indicating a more dramatic metabolic alteration of MEKIs to antigen-stimulated CAR-T cells (Fig. 6d, e, Supplementary Fig. 8e and Supplementary Table 2). Consistent with upregulation of PIK3IP1 and downregulation of “mTORC1 signaling” gene sets, trametinib downregulated genes (*PIK3CB*, etc.)^32^ in PI3K/Akt/mTOR pathway, implying a crosstalk between MAPK and PI3K/Akt/mTOR pathway^33^ (Fig. 6e and supplementary Table 2).

### Single-cell transcriptome analysis of CAR-T cells after MEK inhibition

**Fig. 7:**
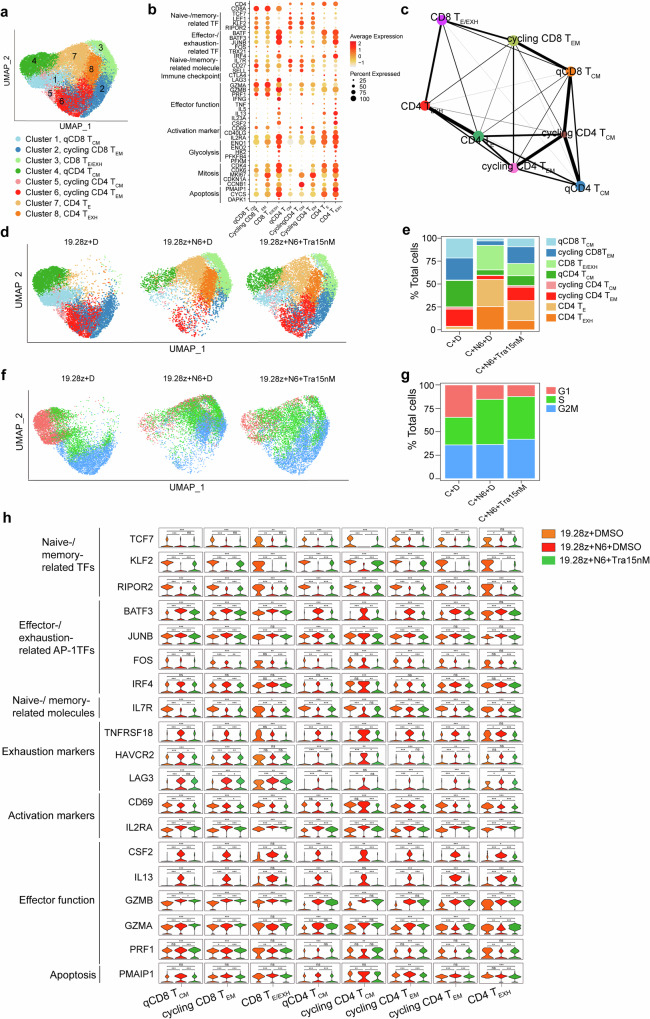
Single-cell transcriptome analysis reveals an increase of memory CAR-T cells while a decrease of effector/exhausted CAR-T cells after MEK inhibition. **a** The UMAP visualization of 35469 cells from all the samples. 8 clusters are indicated by different colors. **b** Dot plot illustrating the expression of the marker genes in different clusters. **c** PAGA analysis shows the potential developmental connectivity among all eight clusters. **d**, **e** The UMAP and bar plots show the constitution of the eight clusters in the 3 samples. **f**, **g** The UMAP and bar plots show the relative frequency of 19.28z CAR-T cells in each phase of the cell cycle. **h** The violin plots depict the single-cell expression of the selected genes in each cluster of the three samples. C 19.28z CAR-T, N6 Nalm-6., D DMSO, Tra trametinib. Q, quiescent. Statistical test was a two-tailed Wilcoxon test. *P< 0.05, **P <0.01, ***P < 0.001, ns: not significant

To elucidate MEKIs’ impacts on individual CAR-T cell transcriptome, we performed single-cell transcriptome analysis on flow-sorted resting CAR-T and Nalm-6-stimulated CAR-T cells treated with DMSO or trametinib. DEGs were identified among the three groups, strongly correlating with bulk RNA-seq findings (Supplementary Fig. 9a). Next, eight clusters were identified and visualized by uniform manifold approximation and projection (UMAP) (Fig. 7a), with full names and identifying markers shown in Methods and Fig. 7b.

Partition-based graph abstraction (PAGA)^34^ constructed developmental trajectories for these clusters, revealing that qCD4 T_CM_ and qCD8 T_CM_ were at the start of CD4 and CD8 CAR-T cells’ developmental trajectories (Fig. 7c), respectively, and their percentage was the most in resting CAR-T cells (Fig. 7c–e). After antigen stimulation, qCD4 T_CM_ could differentiate into cycling CD4 T_CM_ and cycling CD4 T_EM_, indicating the self-renewal and committed differentiation ability of T_CM_^35^ (Fig. 7c), and then cycling CD4 T_EM_ would differentiate into CD4 T_E_ and CD4 T_EXH_. CD8 CAR-T cells’ developmental trajectories were similar to CD4 CAR-T cells, both involving the development of T_CM_ to T_EM_ to T_E/EXH_, with the only difference that there was no cycling CD8 T_CM_ in CD8 CAR-T cells’ developmental trajectories (Fig. 7c).T cell exhaustion and glycolysis scores, AP-1 TFs^22^ (*BATF*, *BATF3*, *JUNB*, and *IRF4*), and glycolysis-related enzymes (*HK2* and *ENO1*, etc.) upregulated as CAR-T cells developed into effector/exhausted state (Fig. 7b and Supplementary Fig. 9b), demonstrating effector/exhausted T cells’ metabolic shift to glycolysis^35^ and the role of AP-1 TFs in driving T cell exhaustion.^22^ Cell division scores revealed qCD8 T_CM_ and qCD4 T_CM_ were the most quiescent with the lowest score, while cycling CD8 T_EM_ and cycling CD4 T_EM_ were the most active in cell division with the highest score (Supplementary Fig. 9b). The variation tendency of exhaustion, glycolysis, and cell-division scores follows our cell cluster definition.

Resting CAR-T cells mainly contained qCD8 T_CM_, cycling CD8 and CD4 T_EM_, and qCD4 T_CM_ (Fig. 7d, e). Upon antigen stimulation, qCD8 T_CM_ and cycling CD8 T_EM_ differentiated into CD8 T_E/EXH_, while qCD4 T_CM_ and cycling CD4 T_EM_ differentiated into CD4 T_E_ and CD4 T_EXH_ (Fig. 7d, e). We found that trametinib restricted antigen-elicited CAR-T cell differentiation, maintaining the pool of qCD8 T_CM_, cycling CD8 and CD4 T_EM_, and qCD4 T_CM_ that can generate T_E_ consistently (Fig. 7d, e). Cell-cycle analysis revealed that clusters 1 and 4 were in G1 and S phase, clusters 2, 5, and 6, with MKI67 expression, were mainly in G2M phase, and clusters 3, 7, and 8 were in S and G2M phase (Fig. 7f). Upon antigen stimulation, trametinib magnified proportion of CAR-T cells in G2M phase (Fig. 7f, g), partially explaining why trametinib promoted antigen-stimulated CAR-T cell proliferation (Fig. 4h and supplementary Fig. 6g).

Further, we identified trametinib-induced cell type-specific transcriptomic change. Trametinib increased naive-/memory-related TFs (*KLF2* and *RIPOR2*),^36^ while reduced AP-1 TFs (*BATF3*, *JUNB*, and *IRF4*), cytokines (*IL13* and *CSF2*), a pro-apoptotic gene (*PMAIP1)*, exhaustion (*TNFRSF18*) and activation markers (*CD69*) in all clusters (Fig. 7h). Besides, after trametinib treatment, TCF7 was upregulated in qCD4 T_CM_, cycling CD4 T_CM_, and CD4 T_EFF_ (Fig. 7h), IL7R was upregulated in clusters except for cycling CD4 T_CM_ and Fos was downregulated in clusters except qCD8 T_CM_, HAVCR2 (TIM-3) was downregulated in clusters except CD8 T_E/EXH_ and cycling CD4 T_EM_, and LAG-3 was downregulated in clusters except qCD8 T_CM_ and cycling CD4 T_CM_ (Fig. 7h). Unlike bulk RNA-seq where all effector molecules were downregulated by trametinib, single-cell RNA-seq revealed upregulation of cytotoxicity molecules (*GZMB*, *GZMA*, and *PRF1*) in certain clusters (Fig. 7h and Fig. 6e). This partly explained why trametinib didn’t affect the cytotoxicity of 19.28z CAR-T cells and even enhanced cytotoxicity in exhaustion-prone GD2.28z CAR-T cells (Fig. 4o and supplementary Fig. 1h, i).

Finally, we identified DEGs in CD8 T_E/EXH_ and CD4 T_EXH_ by comparing trametinib and DMSO-treated groups. 462 upregulated genes were shared between CD8 T_E/EXH_ and CD4 T_EXH_, including mitosis-promoting genes (*KIT*, etc.), TCR signaling suppressors (*PTPN22*, etc.), naive-/memory-related TFs (*LEF1*, etc.) and surface markers (*IL7R*, etc.), effector molecules (*PRF1*, etc.) and *CD28* (Supplementary Fig. 9c). 259 downregulated genes were shared between CD8 T_E/EXH_ and CD4 T_EXH_, including effector/exhaustion-related TFs (*ZBTB32*, etc.), AP-1 TFs (*Fos* and *JunB*, etc), an anergy TF (*EGR2*), activation markers (*IL2RA*, etc.), MAP kinase suppressors (*DUSP2*, etc), cytokine and cytokine receptors (*IL12RB*, etc.), translation-related molecules (*WARS*, etc.), an inhibitory receptor (*TNFRSF18*), and pro-apoptotic genes (*FASLG*, etc.) (Supplementary Fig. 9c). GSEA indicated trametinib upregulated naive-/memory-related genes in both clusters, and “MITOTIC SPINDLE” gene sets in CD8 T_E/EXH_ (Supplementary Fig. 9d), while downregulating Myc- and pERK-regulated, T cell activation/effector/exhaustion-, NFAT pathway-, cytokine signaling-, translation-, mitochondria-, and apoptosis-related genes in both clusters (Supplementary Fig. 9d).

Together, trametinib prevented CAR-T cells from differentiation and reinvigorated exhausted CAR-T cells’ transcriptome by enhancing naive/memory-, cytotoxicity-, and mitosis-related gene expression while diminishing exhaustion-, anergy-, activation-, cytokine-, translation-, mitochondria-, and apoptosis-related genes expression.

### C-Fos and JunB overexpression partially counteracts MEK inhibition

AP-1 TFs influence T cell exhaustion and effector significantly.^22^ Among the AP-1 TFs downregulated by trametinib, c-Fos and JunB are direct downstream targets of MAPK signaling.^37^ C-Fos and c-Jun form classic AP-1 TF, transactivating genes involved in T cell effector^37^ and exhaustion,^38^ while JunB forms immunoregulatory AP-1 complexes (AP-1i) with BATF/BATF3 and IRF4 to drive exhaustion-related gene expression.^22^ Since our transcriptomic data revealed that AP-1 TFs downregulated after MEK inhibition, we hypothesized that c-Fos and JunB downregulation could contribute to MEKIs effects.

**Fig. 8:**
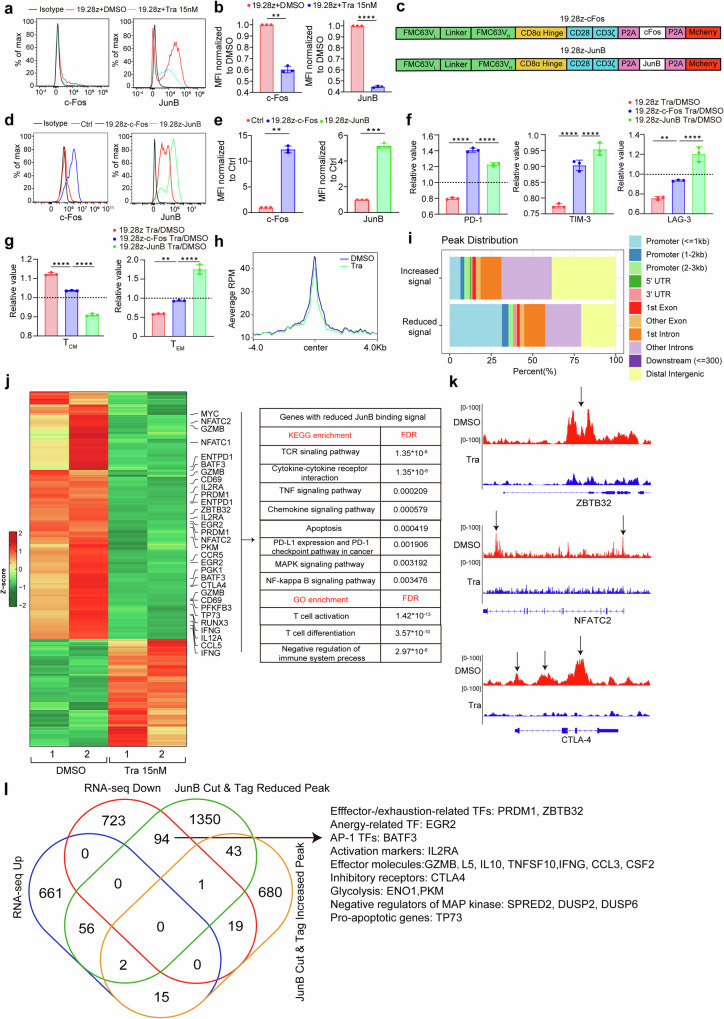
Overexpression of c-Fos and JunB in CAR-T cells partially abrogates the role of MEK inhibition. **a**, **b** Expression of c-Fos and JunB in DMSO- and trametinib-treated CAR-T cells. The histograms (**a**) show data from one representative donor. The expression level of c-Fos and JunB was normalized to DMSO-treated CAR-T cells in (**b**) (*n* = 3). **c** The schematics of 19.28z-c-Fos and 19.28z-JunB CAR expression vector. **d**, **e** c-Fos and JunB expression in Ctrl and c-Fos or JunB 19.28z CAR-T cells. The expression level of c-Fos and JunB was normalized to Ctrl CAR-T cells in (**e**) (*n* = 3). **f**, **g** Flow cytometric analysis of exhaustion markers (**f**) and differentiation state (**g**) of CAR-T cells (*n* = 3). Data are normalized to DMSO-treated group. **h** Enrichment plot of JunB Cut & Tag signal in DMSO- or trametinib-treated 19.28z CAR-T cells. The *x*-axis and *y*-axis show the distance from the center of JunB-bound site and the average RPM across replicates for JunB Cut & Tag, respectively. **i** The peak distribution on the whole genome of genes gaining JunB binding or losing JunB binding after MEK inhibition. **j** Left panel: heatmap of differential peaks identified by Cut & Tag between DMSO- and trametinib-treated CAR-T cells (*n* = 2). Right panel: GO and KEGG enrichment of genes losing JunB binding after MEK inhibition. **k** Representative Cut & Tag sequencing tracks showing binding of JunB to ZBTB32, NFATC2 (encoding NFAT1), and CTLA4 in DMSO- and trametinib-treated CAR-T cells. Arrows denote significant peaks. **l** Venn diagram displayed overlap between DEGs and differential peaks comparing Nalm-6 stimulated 19.28z CAR-T cells treated with trametinib versus DMSO. Error bars are means ± SEM. Statistical tests used were Welch’s *t* test (**b**, **e**) and ordinary one-way ANOVA with Dunnett’s multiple comparison test (**f**, **g**). **P < 0.01, ***P < 0.001, ****P < 0.0001. Tra trametinib

Flow cytometry confirmed c-Fos and JunB downregulation in trametinib-treated CAR-T cells (Fig. 8a, b). Then, we overexpress c-Fos and JunB in CAR-T cells, respectively (Fig. 8c–e). After 9 days of culture, we treated control CAR-T, c-Fos-CAR-T, and JunB-CAR-T cells with trametinib for three days. We normalized PD-1, TIM-3, and LAG-3 positive rates and T_EM_/T_CM_ percentages of trametinib-treated Ctrl, c-Fos, and JunB-CAR-T cells to those of their DMSO-treated counterparts. Overexpressing c-Fos and JunB abrogated trametinib’s role in reducing PD-1, TIM-3, LAG-3, CD69, and differentiation (Fig. 8f, g and Supplementary Fig. 9e), supporting our hypothesis.

Finally, considering that JunB’s rescue role to MEK inhibition was stronger than Fos (Fig. 8f, g), we performed JunB-directed genome-wide Cut & Tag assay in Nalm-6-stimulated CAR-T cells treated with DMSO or trametinib to identify JunB-regulated genes contributing to MEKIs effects. Globally, Cut & Tag profiles indicated decreased JunB binding to DNA after MEK inhibition (Fig. 8h). Peaks with reduced signal were mainly distributed in promoters, while those with increased signal were mainly distributed in the first intron, other introns, and distal intergenic regions (Fig. 8i), implying decreased JunB-driven gene expression after MEK inhibition. Specifically, peaks with reduced signal were observed at gene loci including effector/exhaustion/anergy-related TFs (*BATF3, MYC, NFATC1* and *EGR2*, etc.), exhaustion (*ENTPD1* and *CTLA4*) and activation (*IL2RA* and *CD69*) markers, effector molecules (*GZMB* and *IFNG*, etc.), MAP kinase suppressors (*DUSP2* and *DUSP6*, etc.), glycolysis-related enzymes (*PKM* and *PGK1*, etc.) and a pro-apoptotic gene *TP73* (Fig. 8j, k and Supplementary Fig. 9f). KEGG and GO analysis enriched these genes in T cell activation/differentiation/effector, MAPK and NF-kappa B signaling, immune checkpoint, and apoptosis (Fig. 8j). Among these genes, *BATF3, PRDM1, ZBTB32, EGR2, CTLA4, IL2RA, GZMB, IFNG, ENO1, PKM, SPRED2, DUSP2, DUSP6* and *TP73* showed reduced expression in Nalm-6 stimulated CAR-T cells treated with trametinib (Fig. 8l). Collectively, our data demonstrated that MEKIs’ effects in reducing exhaustion induced by tonic signaling and antigen stimulation partially relied on diminished c-Fos and JunB expression (Supplementary Fig. 10).

## Discussion

Given the significance of MEKIs in targeted therapy^1^ and the roles of MAPK signaling in T cells,^12,13^ it is crucial to understand the influence of MEKIs on CAR-T cells and answer how to combine them to treat cancer. Here, we present a translational strategy to enhance CAR-T cells’ efficacy by incorporating MEKIs during CAR-T cell manufacturing or in-vivo administrating MEKIs.

The effectiveness of MEKIs is closely linked to the exhaustion level and the CD4/CD8 subsets of CAR-T cells. Previous studies have demonstrated that scFv targeting GD2, particularly the scFv derived from 14g2a-E101K, induces the highest tonic signaling, causing GD2 CAR-T cells to be more prone to exhaustion than CD19 CAR-T cells.^6^^,^^22^ Consequently, MEKIs facilitated the proliferation and killing potential of bulk GD2 CAR-T cells (Supplementary Figs. 1i, 3l) and had a more pronounced positive impact on the effector function of CD8 GD2.28z CAR-T cells (Fig. 1j, k and Supplementary Fig. 2f, h). In contrast, scFv targeting CD19 induces the lowest tonic signaling.^6,10^ Hence, incorporating MEKIs into the manufacturing of CD19 CAR-T cells slightly inhibited their proliferation and had no impact on their bulk cytotoxicity (Fig. 1f and Supplementary Fig. 1h). However, when CD19 CAR-T cells are repetitively stimulated by tumor antigen and get into functional exhaustion, MEKIs can promote their proliferation and augment their killing ability (Fig. 4l–n). It has been reported that CD8 CAR-T cells are more prone to exhaustion and apoptosis than CD4 CAR-T cells.^39–41^ Therefore, while MEK inhibition reduces the killing and cytokine release of CD4 CAR-T cells, it protects CD8 CAR-T cells from exhaustion and enhances their killing ability, cytokine release, and percentage both in vivo and in vitro. We speculate that trametinib does not influence the cytotoxicity of bulk 19.28z CAR-T cells because it reduces the cytotoxicity of CD4 CAR-T cells but increases the cytotoxicity of CD8 CAR-T cells.

The in vitro cytotoxicity of CAR-T cells is largely attributed to their ability to release effector cytokines, while the in-vivo efficacy of CAR-T cells is mainly related to their persistence.^3,5,35^ One determining factor of CAR-T cell persistence is their memory/effector differentiation status.^35^ Notwithstanding effector differentiation is helpful to produce effector molecules, it is at the cost of sacrificing the persistence and fitness of CAR-T cells.^35,42^ Trametinib-treated CAR-T cells contained more central memory CAR-T cells, which are more persistent in vivo than effector memory and effector CAR-T cells. Thus, the in vivo efficacy of Trametinib-treated CAR-T cells is better than that of DMSO-treated CAR-T cells. Less cytokine expression in the bulk RNA seq data aligns with the phenotype that MEKIs restricted the differentiation of CAR-T cells towards effector memory and effector states, contributing to the enhanced antitumor activities in vivo. Additionally, our data indicated that although MEK inhibition restrained the production of effector molecules in bulk CAR-T cells, it increased effector function in CD8 CAR-T cells (Fig. 1j, k and Supplementary Fig. 2f, h) and certain clusters defined in single-cell RNA-seq (Fig. 7h). The improved function of these clusters may also contribute to the enhanced antitumor activities in vivo.

Mechanistically, MEKIs downregulate c-Fos and JunB, further repressing genes implicated in T cell effector/exhaustion/differentiation/anergy, glycolysis, and apoptosis. Lynn et al. described that despite oncogenic transformation risk, c-Jun overexpression in CAR-T cells reduced exhaustion by decreasing AP-1i TFs.^22^ In our study, without risk of gene editing, inhibiting MAPK signaling downregulated components of AP-1i TFs (IRF4, JunB, and BATF3). Cut & tag data suggests BATF3 as a downstream target of JunB. Additionally, c-Fos induces PD-1 expression by directly binding to its promoter,^38^ implying that downregulating c-Fos potentially contributes to PD-1 reduction after MEK inhibition. Moreover, we observe the downregulation of NFAT-driven exhaustion-related TFs NR4A^43,44^ and TOX,^45–47^ but not NFAT. Since IRF4 cooperates with BATF and NFAT to drive T cell exhaustion^48^ and NFAT binds to c-Jun, c-Fos, JunB, and Fra-1 to drive gene expression,^49^ we speculate that lack of IRF4, c-Fos, and JunB cooperation may reduce NFAT-driven exhaustion-related TFs, which warrants further investigation.

Furthermore, MEK inhibition downregulated anabolic metabolism-related genes involved in glycolysis, fatty acid synthesis, amino acid metabolism, translation, polyamine synthesis, and nucleotide metabolism, suggesting impaired anabolic metabolism in CAR-T cells. Consistently, prior studies indicated that inhibiting anabolic metabolism through repressing PI3K/Akt/mTOR, MAPK, and Myc pathway,^35^ extends T cell longevity.^35^ While ERK phosphorylation enhances T cells glycolysis by activating Myc,^50^ we didn’t observe Myc downregulation. Instead, we noticed Mxd4 upregulation, which antagonizes Myc. Recent studies proved CDK4/6 inhibition favours memory formation by upregulating Mxd4^24^ and Mxd4 suppresses Myc-dependent T cell death,^51^ implying Mxd4 upregulation probably promotes memory formation and metabolic shift following MEK inhibition. Besides, genes in mTORC1 signaling are downregulated probably because MAPK pathway can cross-activate PI3K/AKT/mTOR pathway.^33^

The study is limited by the absence of clinical evaluation of MEKIs and CAR-T cells combination. We recommend incorporating MEKIs into the culture of clinical CAR-T cell products to improve efficacy. This suggestion is consistent with studies demonstrating that ex vivo small molecule inhibitor treatment improves CAR-T cell function.^52–56^ Administrating MEKIs in vivo probably benefits patients receiving CAR-T therapy by modulating suppressive tumor microenvironment^29^ and reducing CAR-T cells exhaustion and differentiation, particularly those with RAS/RAF-mutated tumors like melanoma, colorectal and breast cancer.^1^ The recommended dose of trametinib is 2 mg per day.^1^ According to the guide for dose conversion between animals and humans,^57^ the corresponding mice dose is 0.4 mg/kg, close to the dose used in our animal studies. Therefore, we suggest that future clinical studies start with a 2 mg dose to investigate the combined effects of trametinib and CAR-T therapy. To avoid the potential inhibitory roles of trametinib in CAR-T cells, we recommend administering trametinib after several days of CAR-T infusion. By this time, CAR-T cells will have become exhausted, and administering trametinib will likely reverse their exhaustion and amplify their function. Our preclinical studies produce the unmet need to evaluate the safety and efficacy of combining MEKIs with CAR-T therapy in future clinical trials.

Overall, our research elucidates the role and mechanisms of MEKIs in counteracting CAR-T cells’ exhaustion and differentiation. These phenotypic and mechanistic insights broaden the utility of MEK inhibitors as immune regulators and pave the way for evaluating the synergy of CAR-T therapy and MEKIs in future clinical trials.

## Materials and methods

### Ethics statement

Animal experiments were performed according to protocols approved by the Ethics Committee of the First Affiliated Hospital, School of Medicine, Zhejiang University. Peripheral blood mononuclear cells (PBMCs) were isolated from the peripheral blood donated by healthy donors via density gradient centrifugation under the protocols approved by the Ethics Committee of the First Affiliated Hospital, College of Medicine, Zhejiang University. All the donors gave written informed consent.

### Cell lines

HEK293T cells and 143B cells were purchased from the National Collection of Authenticated Cell Cultures (Shanghai, China) and MeisenCTCC (Hangzhou, China), respectively. Nalm-6 cells were a gift from iCarTab (Suzhou, China). Nalm-6 cells and 143B cells were engineered to express firefly luciferase and ZsGreen by lentiviral vector packaged using the plasmid vectors (pHIV-Luc-ZsGreen, Catalog no.39196) purchased from Addgene. The engineered Nalm-6 and 143B cells were named Nalm-6-GL and 143B-GL, respectively. Following lentiviral transduction, cells were flow-sorted gating for ZsGreen by Beckman moflo Astrios EQ to achieve >90% purity. All the cell lines were tested by PCR to confirm mycoplasma was negative by use of the sense primer (5′-GGGAGCAAACAGGATTAGATACCCT-3′) and antisense primer (5′-TGCACCATCTGTCACTCTGTTAACCTC-3′) as described in the reference.^58^ The primary Nalm-6 cells and the Nalm-6-GL cells were expanded in RPMI 1640 medium (Corning, Catalog no.10-040-CVR). HEK 293 T cells were cultured in DMEM (Corning, Catalog no.10-013-CMR) medium. All the culture mediums were supplemented with 1% penicillin-streptomycin (Sigma) and 10% FBS (Corning). All the cell lines were cultured at 37 °C in a humidified incubator with 5% CO_2_.

### Mice

Xenograft studies were conducted using female NOD/ShiLtJGpt-Prkdc^em26Cd52^Il2rg^em26Cd22^/Gpt (NCG) mice, which were aged 6–14 weeks and purchased from GemPharmatech Co, Ltd (Nanjing, China). Mice were bred at Animal Facility at Zhejiang Academy of Medical Sciences in pathogen-free conditions.

### Construction of chimeric antigen receptors (CAR) genes and packaging of lentiviral vectors

The single-chain variable fragment (scFv) sequence specific for CD19 and GD2 was derived from Clone FMC63^59^ and 14g2a-E101K,^22^ respectively. 19.28z and GD2.28z CARs were made up of scFv, CD8 hinge, CD28 transmembrane domain, and CD28 and CD3ζ intracellular signaling domains. 19.4-1BBz CAR was made up of scFv, CD8 transmembrane domain, and 4-1BB and CD3ζ intracellular signaling domains. The CAR and mcherry sequence, separated by a P2A sequence, were synthesized and then subcloned into a lentiviral backbone. For c-Fos and JunB single overexpression vectors, codon-optimized cDNAs encoding c-Fos (FOS)-P2A and JunB-P2A were synthesized and subcloned into 19.28z CAR vectors on the site between CAR and mcherry to create CAR-P2A-FOS-MCHERRY and CAR-P2A-JUNB-MCHERRY lentiviral vectors, respectively. HEK 293T cells were transfected with the plasmid encoding CAR and packaging plasmids (psPAX2 and pMD2.G) by PEI (Polysciences, Catalog no.23966-1) transfection. The lentivirus supernatant was collected 48 and 72 h after transfection and concentrated by ultracentrifugation for 2 h at 60,000 g and 4 °C.

### Isolation of T cells and manufacturing of CAR-T cells

Bulk T cells, CD8 T cells and CD4 T cells were separated from PBMCs by EasySep™ Human T Cell Isolation Kit (Stem Cell, Catalog no.17951), EasySep™ Human CD8 + T Cell Isolation Kit (Stem Cell, Catalog no. 17953) and EasySep™ Human CD4 + T Cell Isolation Kit (Stem Cell, Catalog no. 17952), respectively. They were stimulated by CD3/CD28 CTS™ Dynabeads™ (Life Technologies, Catalog no.40203D) at a 3:1 bead-to-T-cell ratio with IL-2 (Peprotech, Catalog no.200-02) 100 U/ml. The day when T cells were activated by CD3/CD28 CTS™ Dynabeads was denoted as day 0. After T cells were activated for 24 h, the lentivirus was added into the cell culture at a multiplicity of infection (MOI) of 3 to 5 in the presence of 6 µg/mL polybrene (Sigma-Aldrich, Catalog no.H9268) to transduce T cells. Four to five days after transduction, anti-CD3/CD28 Dynabeads were removed and CAR-T cells were flow sorted gating for mcherry by Moflo Astrios EQ (Beckman) or FACS AriaII (BD Bioscience) to achieve >90% purity. CAR-T cells were cultured in RPMI1640 medium containing 10% FBS and 100 U/ml IL-2. The culture medium was removed and replenished with fresh medium containing 10% FBS and 100 U/ml IL-2 every 2 days or when it became yellow.

### Flow cytometry

All samples were tested by the CytoFLEX or CytoFLEX Lx Flow Cytometer (Beckman Coulter) and data were analyzed using FlowJo software (Tree Star). Sorting assays were performed using Moflo Astrios EQ (Beckman) or FACS AriaII (BD Bioscience). For surface staining, cells were incubated with antibodies at 4 °C for 25 min in FACS buffer (PBS + 2% FBS). The fluorochrome-labeled anti-human antibodies used for surface staining were: CD3-PE/Cy7 (Biolegend, Catalog no.300420), PD-1 APC (Biolegend, Catalog no.329908), PD-1 BV421 (Biolegend, Catalog no.329920), TIM-3 PE (Biolegend, Catalog no. 345006), LAG-3 PE-Cy7 (Biolegend, Catalog no.369310), LAG-3 APC (Biolegend, Catalog no.369212), CD25 APC (Biolegend, Catalog no.302610), CD25 BV421 (Biolegend, Catalog no.302630), CD69 PE-Cy7 (Biolegend, Catalog no.310912), CD69 APC (Biolegend, Catalog no.310910), CD45RO APC (Biolegend, Catalog no.304210), CD62L PE (Biolegend, Catalog no.304806), CD8 PE-Cy7 (Biolegend, Catalog no.344712), CD8 BV785 (Biolegend, Catalog no.344740), CD4 APC-Cy7 (Biolegend, Catalog no.300518), CD19 APC (Biolegend, Catalog no.302212). The isotype ctrl antibodies used were: PE Mouse IgG1, k Isotype ctrl antibody (Biolegend, Catalog no.400112), APC Mouse IgG1, κ Isotype ctrl antibody (Biolegend, Catalog no.400119), PE/-Cy7 Mouse IgG1, κ Isotype ctrl antibody (Biolegend, Catalog no.400125), BV421 Mouse IgG1, κ Isotype ctrl antibody (Biolegend, Catalog no.400157). Anti-mouse TER119 BV510 antibody (Biolegend, Catalog no.116237) was used to exclude red blood cells for flowcytometric test of CAR-T cells in bone marrow. The expression of CAR was detected by Goat Anti-Mouse IgG, F(ab’)2 fragment (Jackson, Catalog no.115-066-006) and streptavidin-PE (BD, Catalog no.554061). Annexin V APC (Biolegend, Catalog no.640941) was used to evaluate the percentage of apoptotic CAR-T cells. Zombie Aqua™ Fixable Viability Kit (Biolegend, Catalog no.423102) was used to exclude dead cells. Intracellular staining of c-Fos and JunB was performed after CAR-T cells were treated with trametinib for 3 days. The cells were stained by Fixable Viability Dye (eBioscience, Catalog no.65-0867-14) to exclude dead cells and then fixed with 4% formaldehyde, followed by permeabilization by ice-cold methanol. PE-conjugated anti-rabbit IgG (H + L) (CST, Catalog no.79408) was used as the secondary antibody following staining with the rabbit anti-c-Fos (CST, clone 9F6) and rabbit anti-JunB (CST, clone C37F9) antibody.

### CAR-T cell counting

For in-vitro experiments, the number of CAR-T cells in each group was calculated by multiplying the percentage of CAR-T cells and the number of viable cells together, where the number of viable cells was counted with trypan blue using Countstar® BioTech Automated Cell Counter (ALIT Life Science) and the percentage of CAR-T cells was determined by flow cytometry. For in-vivo experiments, we infused flow-sorted CAR-T cells (purity >90%) into mice, and the bone marrow of the four limbs or solid tumor samples were collected and run out to be analyzed in flow cytometry so that the total number of human CD3 positive events can reflect the number of CAR-T cells.

### Cell proliferation assay

CellTrace™ Violet Cell Proliferation Kit (C34557) was purchased from ThermoFisher.

On day 6 of in-vitro culture, CAR-T cells were labeled with CellTrace™ Violet following the manufacturer’s instructions. Subsequently, CAR-T cells were cultured with MEKIs or DMSO for six days. CellTrace™ Violet dilution was assessed on day 12 by flow cytometry.

### Evaluation of the impacts of MEKIs on the in-vitro culture of CAR-T cells

The MEK inhibitors used in the study were trametinib (Selleck, Catalog no.S2673), cobimetinib (Selleck, Catalog no.S8041), and binimetinib (Selleck, Catalog no.S7007). On day 6 of in-vitro culture, CAR-T cells were used for experiments. Briefly, CAR-T cells were cultured with MEK inhibitors or DMSO for 9 days till day 15. Every 3 days, the number of CAR-T cells was counted, and the culture medium and the inhibitors were replenished. Flow cytometry was utilized to evaluate the phenotype of CAR-T cells on day 9 and day 15 by gating on mcherry-positive cells. For animal experiments, the CAR-T cells treated with MEK inhibitors or DMSO were collected for adoptive transfer on day 15. For RNA sequencing and western blot, flow-sorted CAR-T cells were cultured with trametinib or DMSO from day 6 to day 15. On day 15, the RNA and protein of CAR-T cells were extracted and used for the assay.

### Stimulation of CAR-T cells by Nalm-6 cells

CAR-T cells cultured in vitro for 9 to 14 days were used for experiments. 1 * 10^6^ Nalm-6 cells and 1 * 10^6^ flow-sorted CAR-T cells per well were cocultured in 6-well plates. Trametinib at indicated concentrations or DMSO was added at the beginning of coculture. After 72 h, the RNA and protein of CAR-T cells were extracted for RNA sequencing and western blot, respectively. The flowcytometric phenotype, cell number, and cytotoxicity of CAR-T cells were evaluated using resting CAR-T cells and Nalm-6-stimulated CAR-T cells as controls (see schematic diagram in the upper panel of Fig. 4a).

For some experiments, 2 * 10^6^ Nalm-6 cells and 2 * 10^6^ flow-sorted CAR-T cells were cocultured in T25 flasks for 48 h. After 48 h, flow cytometry was applied to detect the phenotype change of CAR-T cells and determine that Nalm-6 cells had been completely eradicated. Then, 1 * 10^6^ stimulated CAR-T cells per well were cultured in 6-well plates with trametinib or DMSO. After 72 h, the flowcytometric phenotype, cell number, and cytotoxicity of CAR-T cells for each well were assessed using CAR-T cells treated by DMSO as control (see schematic diagram in the middle panel of Fig. 4a)

For repeated target antigen exposure assay, 5 * 10^5^ Nalm-6 cells and 5 * 10^5^ flow-sorted CAR-T cells were cocultured in 12-well plates with trametinib or DMSO. Every 24 h, the number of CAR-T cells was counted, the equal number of Nalm-6 cells was rechallenged, and the culture medium containing trametinib or DMSO was replenished. After 3-time exposure to the target antigen, the CAR-T cells were used for cytotoxicity assay in the culture medium free of trametinib or DMSO (see schematic diagram in the lower panel of Fig. 4a).

### Cytotoxicity assay

100 μl Nalm-6-GL cells or 143B-GL cells were cultured in 96–well round bottom plates at 2 × 10^5^ cells/ml concentration in triplicate wells. CAR-T cells or UTD cells at the same concentration were added at indicated effector-to-target (E:T) ratios and incubated at 37 °C for 18 or 24 h, respectively. At the end of the assay, cell extracts were created and substrate for the luciferase was added using the Bright-Glo Luciferase Assay System (Promega Corporation, Catalog no.E2620) according to the manufacturer’s instructions. The luminescence produced by the luciferase was measured by a SpectraMax M5 microplate reader (Molecular Devices). The lysis rate was calculated by the following equation: lysis rate (%) = (Vt-Vcar-t)/Vt × 100%. Vt was the luminescence value of the wells where UTD cells were added and Vcar-t was the luminescence value of the wells where CAR-T cells were added. For the cytotoxicity assay in Fig. 4o, a tumor-only group was set, and the lysis rate was calculated by the following equation: lysis rate (%) = (Vtumor-Vt/car-t) / Vtumor × 100%. Vtumor was the luminescence value of the wells containing only tumor cells. Vt/car-t was the luminescence value of the wells where CAR-T or UTD cells were added.

### Cytokine production

1 × 10^5^ CAR-T cells and 1 × 10^5^ tumor cells were cocultured in 200 μl culture media in 96-well plates for 24 h. Triplicate wells were plated for each condition. Culture supernatants were collected, and the concentration of IFN-γ, IL-2, TNF-α, granzyme B, CCL3, and CXCL8 was measured by Cytometric Bead Array (CBA) kits (BD Biosciences). The data were analyzed by FCAP Array™ (v3.0) software.

### Western blot

1 × 10^6^ cells from control and treated groups were collected, centrifuged at 300 g for 5 min, and washed with PBS, respectively. The cells were subsequently lysed in RIPA (Beyotime Institute of Biotechnology, Catalog no.P0013C) buffer containing protease inhibitor cocktail (A32963, Thermo Fisher Scientific). Protein extracts were centrifuged at 12,000 g for 10 min, and the supernatant was collected and added with 5 × loading buffer, then heated at 100 °C for 10 min. The prepared protein samples were added to SDS-PAGE gel for electrophoresis, transferred to the polyvinylidene difluoride membrane, and blocked with 5% fat-free milk at room temperature for 1–2 h. Then the membranes were incubated with primary antibody overnight at 4 °C, followed by secondary antibodies for 1 h at room temperature, and chemiluminescence detection was carried out using a chemiluminescence imaging system (Clinx Science, Shanghai, China). The primary antibodies anti-Erk1/2 (Catalog no.4695) and anti-Erk1/2 pThr202/Tyr204 (Catalog no.4370) were purchased from Cell Signaling Technology, the reference antibody anti-β-Tublin (Catalog no.HC101) was purchased from TransGen Biotech and the secondary antibody anti-rabbit IgG-HRP (Catalog no.abs20040) was purchased from Absin.

### Bulk RNA extraction and transcriptome sequencing

According to manual instruction, total RNA was extracted from cells using Trizol (ThermoFisher, Catalog no.15596026). Sequencing was performed by Beijing Genomics Institute (BGI), Shenzhen. Briefly, oligo(dT)-attached magnetic beads were used to purify mRNA. Purified mRNA was fragmented and reversely transcribed to first-strand cDNA using random hexamer-primed reverse transcription, followed by second-strand cDNA synthesis. Then, end repair was performed by adding a-Tailing Mix and RNA Index Adapters. Next, the cDNA fragments acquired from the previous step were amplified by PCR, and products were purified by Ampure XP Beads, then dissolved in EB solution. The Agilent Technologies 2100 bioanalyzer was used for quality control of the products. The double-stranded PCR products from the previous step were denatured by heating and circularized by the splint oligo sequence to obtain the final library. The single-stranded circle DNA (ssCir DNA) was formatted as the final library. Phi29 was applied to amplify the final library to generate a DNA nanoball (DNB) that contained more than 300 copies within one molecule. Finally, DNBs were loaded into the patterned nanoarray, and single-end reads containing 50 bases were produced on the BGIseq500 platform (BGI-Shenzhen, China).

### Bulk RNA-seq analysis

Trimmomatic (v0.38) was used to remove poor-quality reads.^60^ The clean data were aligned to the human reference genome (GRCh38) by HISAT2 (v2.1.0)^61^ using default settings. The gene counts were calculated by HTSeq (v0.11.2)^62^ with the parameter “--mode intersection-strict --stranded no --minaqual 1”. The R package edgeR (v3.22.3)^63^ was used to identify differentially expressed genes (DEGs) with a fold change > 2 and false discovery rate (FDR) < 0.05. Principal component analysis (PCA) was conducted using the R package FactoMineR (v2.4).^64^

KEGG pathway enrichment analysis of DEGs was performed by the R package clusterProfiler (v3.12).^65,66^ Gene set enrichment analysis (GSEA) was applied to explore whether gene sets showed significant differences between the two groups,^67^ with a significance of FDR < 0.05.

### Single-cell RNA-seq

Single-cell RNA sequencing of resting CAR-T cells, Nalm-6-stimulated CAR-T cells, and trametinib-treated Nalm-6-stimulated CAR-T cells was performed by Berry Genomics (Beijing) via the Chromium Single Cell 3’ Library & Gel Bead Kit v3 (10X Genomics) according to the manufacturer’s instructions. Briefly, Dead cells were removed using a Dead Cell Removal kit (Miltenyi Biotec, Catalog no.130-090-101). Then cells were washed with 0.04% BSA DPBS three times and were resuspended to a concentration of 700 ~ 1200 cells/ul (viability ≥ 85%) as determined using the Countess® II Automated Cell Counter. After that, cells were used to generate single-cell gel beads in an emulsion. Following reverse transcription, gel beads in the emulsion were disrupted, and barcoded complementary DNA was isolated and amplified by PCR. After Enzymatic fragmentation, end repair, and poly A tailing, sample indexes were added and amplified following the manufacturer’s protocol. The final libraries were quality control checked by Agilent 2100 Bioanalyzer and sequenced on a NovaSeq platform (Illumina) to generate 150 bp paired-end Reads to a depth of more than 100,000 reads per cell.

### Single-cell RNA-seq analysis

scRNA-seq raw data was deduplicated and aligned to the human reference genome (GRCh38). The raw count matrix was generated by Cellranger (v3.1) with default parameters and transformed into a Seurat object by the R package Seurat (v3.2).^68^ Genes detected in less than 3 cells were removed. High-quality cells with expressed genes >200, mitochondrial transcript ratio <15%, and UMI number >3000 were retained. The scRNA-seq data was normalized using the NormalizeData function of Seurat with default parameters. PCA was performed using the top 2000 variable genes. The batch effects were eliminated by iteratively corrected PCA embedding using the R package Harmony (v0.99.9).^69^ Cell clustering and nonlinear dimensionality reduction (Uniform Manifold Approximation and Projection, UMAP) were then performed using the first 40 components generated by Harmony.

The cell clusters were annotated based on well-known cell markers. The main cell types were annotated as qCD8 T_CM_ (quiescent CD8 central memory T cells, *CD8A* + *SELL* + *RIPOR2* + *LEF1* + *CD27* + ), cycling CD8 T_EM_ (cycling CD8 effector memory T cells, *CD8A* + *MKI67* + *GZMA* + *PRF1* + ), CD8 T_E/EXH_ (CD8 effector/exhausted T cells, *CD8A* + *BATF3* + *JUNB* + *GZMB* + *IL2RA* + *LAG3* + ), qCD4 T_CM_ (quiescent CD4 central memory T cells_,_ *CD4* + *KLF2* + *TCF7* + *RIPOR2* + *IL7R* + ), cycling CD4 T_CM_ (cycling CD4 central memory T cells_,_ *CD4* + *KLF2* + *SELL* + *MKI67* + *CCNB1* + ), cycling CD4 T_EM_ (cycling CD4 effector memory T cells, *CD4* + *MKI67* + *TNF* + ), CD4 T_E_ (CD4 effector T cells, *CD4* + *IL13* + *BATF* + *IL2RA* + *ENO1* + ) and CD4 T_EXH_ (CD4 exhausted T cells, *CD4* + *JUNB* + *BATF* + *BATF3* + *IRF4* + *PMAIP1* + ). Seurat’s FindAllMarkers function was used to identify cell type-specific genes or genes differentially expressed between groups. scRNA-seq data was visualized using Seurat tools and the R package ggplot2.^70^

The gene sets of HALLMARK_GLYCOLYSIS from MSigDB, cell division (GO:0051301) from the Gene Ontology, and T cell exhaustion-related genes defined by Guo et al.^71^ were used to calculate the glycolysis, cell division, and T cell exhaustion score, respectively. Function AddMouduleScore embedded in Seurat was used with default parameters to calculate the gene signatures of each cell cluster. The average expression matrix of all genes in each gene set was generated and then spliced into 24 bins according to the average expression value. The control gene set was composed of 100 genes randomly selected from each bin. The gene signature score was produced by subtracting the average expression of background genes from the average expression of each gene set. The cell cycle score (G1, S, G2M) was calculated by the CellCycleScoring function in Seurat using a similar method. The pseudotime trajectory was constructed using the partition-based graph abstraction (PAGA) method with scanpy (v1.9.1).^72^

### In-vivo studies

Animal studies were performed with at least 4 mice per group without randomization or blinding. For xenograft experiments using Nalm-6, mice were inoculated with 1 * 10^6^ Nalm-6-GL cells per mouse by tail vein injection. Tumor burden and distribution were assessed by bioluminescence imaging using a Spectrum IVIS instrument (PerkinElmer). The total radiance was calculated in regions of interest that encompassed the whole body of each mouse by Living Image Software (PerkinElmer) after five days of tumor injection. The mice were distributed to each group according to their tumor burden to guarantee the homogeneity of tumor burdens among different groups. After six days of tumor engraftment, flow-sorted CAR-T cells (purity > 90%) cultured in vitro with DMSO or trametinib for 9 days at doses indicated in the corresponding figure legend were infused into mice intravenously using UTD cells as controls. The bone marrow of mice was collected by mechanical dissociation at indicated time points to analyze the phenotype of CAR-T cells. The live imaging was carried out regularly as indicated to assess tumor burden.

For xenograft experiments using 143B cells, 1 × 10^6^ tumor cells were intramuscularly injected into the flank of mice. After 2 days of tumor engraftment, tumor burden was assessed through bioluminescence imaging using a Spectrum IVIS instrument and quantified using Living Image software (Perkin Elmer). The mice were distributed to each group according to their tumor burden to guarantee the homogeneity of tumor burdens among different groups. Flow-sorted 1.3 × 10^7^ CAR-T cells (purity > 90%) were infused intravenously on day 3 after tumor injection. Mice were orally administered with 100 μL vehicle (DMSO plus corn oil) or trametinib at 0.3 mg/kg. Tumors were measured regularly using a digital vernier caliper. The tumor volume was calculated using the formula: V = L × W^2^ × 0.53, where V is the tumor volume, L is the tumor length (longer diameter), and W is the the tumor width (shorter diameter). Mice were euthanized when the tumor volume reached 1.5 cm^3^. For detecting the phenotype of tumor-infiltrating CAR-T cells and the expression of PDL1 in tumor cells, some mice were euthanized on Day 19 post-CAR-T infusion. Fresh tumors were minced and digested in DMEM containing collagenase I (1 mg/mL, Solarbio) and DNase I (200 μg/mL, Roche) for 1 h at 37 °C and filtered through a 0.07 mm cell strainer (Corning).

The red blood cells (RBCs) were removed from bone marrow and solid tumors using RBC lysis buffer (Biolegend). Single-cell suspensions were stained with Fixable Dead Cell Dyes (Biolegend) followed by FcR-Blocker (eBioscience) following the manufacturer’s recommendations.

### Cut & tag

Liquid-nitrogen cryopreserved equal Nalm-6-stimulated CAR-T cells treated with DMSO or trametinib were thawed, and dead cells were removed using EasySep™ Dead Cell Removal Kit (Stem Cell, Catalog no.17899). Then samples were split into two replicates before all subsequent steps. The sequencing library was constructed via Hyperactive Universal CUT&Tag Assay Kit for Illumina (Vazyme) according to the manufacturer’s instructions. Briefly, cells were washed by wash buffer (Vazyme) supplemented with protease inhibitor cocktail (Targetmol) and bound to ConA magnetic beads (Vazyme). ConA-bound cells were suspended in antibody buffer (Vazyme) supplemented with digitonin (Vazyme) for incubation with the rabbit anti-JunB (CST clone C37F9) antibody at 4 °C overnight. Samples were washed to remove unbound antibodies, resuspended, and incubated in Dig-300 buffer (Vazyme) containing pA/G-Tnp (Vazyme), protease inhibitor cocktail, and digitonin at room temperature for 1 h. Then, samples were washed in Dig-300 buffer containing protease inhibitor cocktail and digitonin three times, resuspended in fragmentation buffer, and incubated at 37 °C for 1 h to allow the pA/G-Tnp fragmentation reaction to go to completion. After that, proteinase K (Vazyme), buffer L/B (Vazyme), and DNA extract beads (Vazyme) were added to the fragmented sample and incubated at 55 °C for 10 min. Buffer WA (Vazyme) and buffer WB (Vazyme) were added to wash beads, and the buffer was air dried. Double distilled water (ddH_2_O) was added to elute DNA from beads. CAM (Vazyme) and barcoded i5 and i7 primer (Vazyme) were added to the sample, and PCR amplification was performed using 13 cycles. Cut & Tag libraries were cleaned with VAHTS DNA Clean Beads (Vazyme #N411) and 80% ethanol. After that, ddH_2_O was added to elute DNA from beads. The libraries were quantified by DNF-915(Agilent) and qPCR.

According to the manufacturer’s instructions, the index-coded samples were clustered on a cBot Cluster Generation System using TruSeq PE Cluster Kit v3-cBot-HS (Illumina). The library preparations were sequenced on the Illumina Novaseq platform at Novogene Science and Technology Co., Ltd (Beijing, China), and 150 bp paired-end reads were generated.

### Cut & tag analysis

Adaptors and poor-quality bases were filtered with Trimmomatic (v0.38). Clean data were aligned to human reference genome (GRCh38) using bowtie2 (v2.3.4.1)^73^ with parameters “--very-sensitive-local --no-unal --no-mixed --no-discordant -I 10 -X 2000”. The resulting bam files were converted to bigwig files and visualized in the integrative genomics viewer (IGV; v.2.13). The peaks were called by Macs2 (v.2.1.3.3)^74^ with parameters “--nomodel --shift -100 --extsize 200 --SPMR --keep-dup 1 -q 0.01”. Differential binding regions were determined by R package Diffbind (v2.16).^75^ Peaks were annotated to the nearest genes by the R package ChIPseeker (v1.30.3)^76^ function annotatePeak (Promoter region: TSS ± 3 kb). The average signal of the centers of JUNB-binding sites of each group was calculated and visualized by deepTools (v3.1.3)^77^ with “--binSize 50”.

### Statistics

No statistical methods were applied to predetermine the sample size. All data, except the transcriptome and Cut & Tag data, were analyzed and plotted with GraphPad Prism 8 (GraphPad Software). For experiments involving at least 3 groups, paired or unpaired one-way ANOVA with Dunnett’s multiple comparison test was used to analyze data. For experiments involving 2 groups, paired or unpaired student’s *t* test, Wilcoxon test, Welch’s *t* test or Mann-Whitney test were used to analyze data. For survival analysis of mice, a Kaplan–Meier curve and the log-rank Mantel–Cox test were used to compare survival differences among the groups. All tests were two-sided. *P* < 0.05 was considered statistically significant. The statistical test used for each figure is described in the corresponding figure legend.

## Supplementary information

Supplementary Figures

Supplementary Table 1

Supplementary Table 2

Supplementary table legend

## Data Availability

Raw genomics data from human samples have been deposited at China National Gene Bank, and accession number is CNP0002478. Data will be released on Nov.5th, 2024. Any additional information required to reanalyze the data reported in this paper is available from the corresponding author upon request.

## References

[1] Cheng, Y. & Tian, H. Current development status of MEK inhibitors. *Molecules* **22**, 1551 (2017). 28954413 10.3390/molecules22101551 PMC6151813

[2] Ullah, R., Yin, Q., Snell, A. H. & Wan, L. RAF-MEK-ERK pathway in cancer evolution and treatment. *Semin Cancer Biol.* **85**, 123–154 (2022). 33992782 10.1016/j.semcancer.2021.05.010

[3] Majzner, R. G. & Mackall, C. L. Clinical lessons learned from the first leg of the CAR T cell journey. *Nat. Med.* **25**, 1341–1355 (2019). 31501612 10.1038/s41591-019-0564-6

[4] Zhu, Y. M. et al. Anti-CD19 chimeric antigen receptor T-cell therapy for adult Philadelphia chromosome-positive acute lymphoblastic leukemia: two case reports. *Medicine* **95**, e5676 (2016). 28002337 10.1097/MD.0000000000005676 PMC5181821

[5] Feucht, J. et al. Calibration of CAR activation potential directs alternative T cell fates and therapeutic potency. *Nat. Med.* **25**, 82–88 (2019). 30559421 10.1038/s41591-018-0290-5 PMC6532069

[6] Long, A. H. et al. 4-1BB costimulation ameliorates T cell exhaustion induced by tonic signaling of chimeric antigen receptors. *Nat. Med.* **21**, 581–590 (2015). 25939063 10.1038/nm.3838 PMC4458184

[7] Singh, N. et al. Impaired death receptor signaling in leukemia causes antigen-independent resistance by inducing CAR T cell dysfunction. *Cancer Discov.* **10**, 552–567 (2020). CD-19-0813. 32001516 10.1158/2159-8290.CD-19-0813 PMC7416790

[8] Wang, H., Song, X., Shen, L., Wang, X. & Xu, C. Exploiting T cell signaling to optimize engineered T cell therapies. *Trends Cancer* **8**, 123–134 (2022). 34810156 10.1016/j.trecan.2021.10.007

[9] Kouro, T., Himuro, H. & Sasada, T. Exhaustion of CAR T cells: potential causes and solutions. *J. Transl. Med.* **20**, 239 (2022). 35606821 10.1186/s12967-022-03442-3 PMC9125881

[10] Chen, J. et al. Tuning charge density of chimeric antigen receptor optimizes tonic signaling and CAR-T cell fitness. *Cell Res.* **33**, 341–354 (2023). 36882513 10.1038/s41422-023-00789-0 PMC10156745

[11] Weber, E. W. et al. Pharmacologic control of CAR-T cell function using dasatinib. *Blood Adv.* **3**, 711–717 (2019). 30814055 10.1182/bloodadvances.2018028720 PMC6418502

[12] Kuske, M. et al. Immunomodulatory effects of BRAF and MEK inhibitors: implications for Melanoma therapy. *Pharm. Res.* **136**, 151–159 (2018). 10.1016/j.phrs.2018.08.019 30145328

[13] Verma, V. et al. MEK inhibition reprograms CD8(+) T lymphocytes into memory stem cells with potent antitumor effects. *Nat. Immunol.* **22**, 53–66 (2021). 33230330 10.1038/s41590-020-00818-9 PMC10081014

[14] Tian, J. et al. Combined PD-1, BRAF and MEK inhibition in BRAF(V600E) colorectal cancer: a phase 2 trial. *Nat. Med.* **29**, 458–466 (2023). 36702949 10.1038/s41591-022-02181-8 PMC9941044

[15] Stock, S., Kluever, A.-K., Endres, S. & Kobold, S. Enhanced chimeric antigen receptor T cell therapy through co-application of synergistic combination partners. *Biomedicines* **10**, 307 (2022). 35203517 10.3390/biomedicines10020307 PMC8869718

[16] Gargett, T., Fraser, C. K., Dotti, G., Yvon, E. S. & Brown, M. P. BRAF and MEK inhibition variably affect GD2-specific chimeric antigen receptor (CAR) T-cell function in vitro. *J. Immunother.* **38**, 12–23 (2015). 25415284 10.1097/CJI.0000000000000061

[17] Dorrie, J. et al. BRAF and MEK inhibitors influence the function of reprogrammed T cells: consequences for adoptive T-cell therapy. *Int. J. Mol. Sci.* **19**, 289 (2018). 29346301 10.3390/ijms19010289 PMC5796234

[18] Tomida, A. et al. Inhibition of MEK pathway enhances the antitumor efficacy of chimeric antigen receptor T cells against neuroblastoma. *Cancer Sci.* **112**, 4026–4036 (2021). 34382720 10.1111/cas.15074 PMC8486218

[19] Salter, A. I. et al. Phosphoproteomic analysis of chimeric antigen receptor signaling reveals kinetic and quantitative differences that affect cell function. *Sci. Signal* **11**, eaat6753 (2018). 30131370 10.1126/scisignal.aat6753 PMC6186424

[20] Tran, B. & Cohen, M. S. The discovery and development of binimetinib for the treatment of melanoma. *Expert Opin. Drug Discov.* **15**, 745–754 (2020). 32249628 10.1080/17460441.2020.1746265 PMC7539481

[21] Gilmartin, A. G. et al. GSK1120212 (JTP-74057) is an inhibitor of MEK activity and activation with favorable pharmacokinetic properties for sustained in vivo pathway inhibition. *Clin. Cancer Res.* **17**, 989–1000 (2011). 21245089 10.1158/1078-0432.CCR-10-2200

[22] Lynn, R. C. et al. c-Jun overexpression in CAR T cells induces exhaustion resistance. *Nature* **576**, 293–300 (2019). 31802004 10.1038/s41586-019-1805-z PMC6944329

[23] Chen, Y., Zander, R., Khatun, A., Schauder, D. M. & Cui, W. Transcriptional and epigenetic regulation of effector and memory CD8 T cell differentiation. *Front. Immunol.* **9**, 2826 (2018). 30581433 10.3389/fimmu.2018.02826 PMC6292868

[24] Heckler, M. et al. Inhibition of CDK4/6 promotes CD8 T-cell memory formation. *Cancer Discov.* **11**, 2564–2581 (2021). 33941591 10.1158/2159-8290.CD-20-1540 PMC8487897

[25] Collins, S. et al. Opposing regulation of T cell function by Egr-1/NAB2 and Egr-2/Egr-3. *Eur. J. Immunol.* **38**, 528–536 (2008). 18203138 10.1002/eji.200737157 PMC3598016

[26] Seo, W., Jerin, C. & Nishikawa, H. Transcriptional regulatory network for the establishment of CD8(+) T cell exhaustion. *Exp. Mol. Med.* **53**, 202–209 (2021). 33627794 10.1038/s12276-021-00568-0 PMC8080584

[27] Safford, M. et al. Egr-2 and Egr-3 are negative regulators of T cell activation. *Nat. Immunol.* **6**, 472–480 (2005). 15834410 10.1038/ni1193

[28] Lake, D., Correa, S. A. & Muller, J. Negative feedback regulation of the ERK1/2 MAPK pathway. *Cell Mol. Life Sci.* **73**, 4397–4413 (2016). 27342992 10.1007/s00018-016-2297-8 PMC5075022

[29] Coelho, M. A. et al. Oncogenic RAS signaling promotes tumor immunoresistance by stabilizing PD-L1 mRNA. *Immunity* **47**, 1083–1099 e1086 (2017). 29246442 10.1016/j.immuni.2017.11.016 PMC5746170

[30] Ebert, P. J. R. et al. MAP kinase inhibition promotes T cell and anti-tumor activity in combination with PD-L1 checkpoint blockade. *Immunity* **44**, 609–621 (2016). 26944201 10.1016/j.immuni.2016.01.024

[31] Uche, U. U. et al. PIK3IP1/TrIP restricts activation of T cells through inhibition of PI3K/Akt. *J. Exp. Med.* **215**, 3165–3179 (2018). 30429249 10.1084/jem.20172018 PMC6279406

[32] Zheng, W. et al. PI3K orchestration of the in vivo persistence of chimeric antigen receptor-modified T cells. *Leukemia* **32**, 1157–1167 (2018). 29479065 10.1038/s41375-017-0008-6 PMC5943191

[33] De Luca, A., Maiello, M. R., D’Alessio, A., Pergameno, M. & Normanno, N. The RAS/RAF/MEK/ERK and the PI3K/AKT signalling pathways: role in cancer pathogenesis and implications for therapeutic approaches. *Expert Opin. Ther. Targets* **16**, S17–S27 (2012). 22443084 10.1517/14728222.2011.639361

[34] Wolf, F. A. et al. PAGA: graph abstraction reconciles clustering with trajectory inference through a topology preserving map of single cells. *Genome Biol.* **20**, 59 (2019). 30890159 10.1186/s13059-019-1663-x PMC6425583

[35] Kishton, R. J., Sukumar, M. & Restifo, N. P. Metabolic regulation of T cell longevity and function in tumor immunotherapy. *Cell Metab.* **26**, 94–109 (2017). 28683298 10.1016/j.cmet.2017.06.016 PMC5543711

[36] Froehlich, J. et al. FAM65B controls the proliferation of transformed and primary T cells. *Oncotarget* **7**, 63215–63225 (2016). 27556504 10.18632/oncotarget.11438 PMC5325358

[37] Atsaves, V., Leventaki, V., Rassidakis, G. Z. & Claret, F. X. AP-1 transcription factors as regulators of immune responses in cancer. *Cancers* **11**, 1037 (2019). 31340499 10.3390/cancers11071037 PMC6678392

[38] Xiao, G., Deng, A., Liu, H., Ge, G. & Liu, X. Activator protein 1 suppresses antitumor T-cell function via the induction of programmed death 1. *Proc. Natl. Acad. Sci. USA* **109**, 15419–15424 (2012). 22949674 10.1073/pnas.1206370109 PMC3458364

[39] Wang, D. et al. Glioblastoma-targeted CD4+ CAR T cells mediate superior antitumor activity. *JCI Insight* **3**, e99048 (2018). 29769444 10.1172/jci.insight.99048 PMC6012522

[40] Yang, Y. et al. TCR engagement negatively affects CD8 but not CD4 CAR T cell expansion and leukemic clearance. *Sci. Transl. Med.* **9**, eaag1209 (2017). 29167392 10.1126/scitranslmed.aag1209 PMC6944272

[41] Melenhorst, J. J. et al. Decade-long leukaemia remissions with persistence of CD4(+) CAR T cells. *Nature* **602**, 503–509 (2022). 35110735 10.1038/s41586-021-04390-6 PMC9166916

[42] Gattinoni, L. et al. Acquisition of full effector function in vitro paradoxically impairs the in vivo antitumor efficacy of adoptively transferred CD8+ T cells. *J. Clin. Invest.* **115**, 1616–1626 (2005). 15931392 10.1172/JCI24480 PMC1137001

[43] Chen, J. et al. NR4A transcription factors limit CAR T cell function in solid tumours. *Nature* **567**, 530–534 (2019). 30814732 10.1038/s41586-019-0985-x PMC6546093

[44] Liu, X. et al. Genome-wide analysis identifies NR4A1 as a key mediator of T cell dysfunction. *Nature* **567**, 525–529 (2019). 30814730 10.1038/s41586-019-0979-8 PMC6507425

[45] Seo, H. et al. TOX and TOX2 transcription factors cooperate with NR4A transcription factors to impose CD8(+) T cell exhaustion. *Proc. Natl Acad. Sci. USA* **116**, 12410–12415 (2019). 31152140 10.1073/pnas.1905675116 PMC6589758

[46] Alfei, F. et al. TOX reinforces the phenotype and longevity of exhausted T cells in chronic viral infection. *Nature* **571**, 265–269 (2019). 31207605 10.1038/s41586-019-1326-9

[47] Khan, O. et al. TOX transcriptionally and epigenetically programs CD8(+) T cell exhaustion. *Nature* **571**, 211–218 (2019). 31207603 10.1038/s41586-019-1325-x PMC6713202

[48] Man, K. et al. Transcription factor IRF4 promotes CD8(+) T cell exhaustion and limits the development of memory-like T cells during chronic infection. *Immunity* **47**, 1129–1141 e1125 (2017). 29246443 10.1016/j.immuni.2017.11.021

[49] McGuire, K. L. & Iacobelli, M. Involvement of Rel, Fos, and Jun proteins in binding activity to the IL-2 promoter CD28 response element/AP-1 sequence in human T cells. *J. Immunol.* **159**, 1319–1327 (1997). 9233628

[50] Wei, X. et al. The evolutionarily conserved MAPK/Erk signaling promotes ancestral T-cell immunity in fish via c-Myc-mediated glycolysis. *J. Biol. Chem.* **295**, 3000–3016 (2020). 31996375 10.1074/jbc.RA119.012231 PMC7062195

[51] Vasilevsky, N. A., Ruby, C. E., Hurlin, P. J. & Weinberg, A. D. OX40 engagement stabilizes Mxd4 and Mnt protein levels in antigen-stimulated T cells leading to an increase in cell survival. *Eur. J. Immunol.* **41**, 1024–1034 (2011). 21400495 10.1002/eji.201040449 PMC3575515

[52] Zhang, H. et al. Dasatinib enhances anti-leukemia efficacy of chimeric antigen receptor T cells by inhibiting cell differentiation and exhaustion. *J. Hematol. Oncol.* **14**, 113 (2021). 34289897 10.1186/s13045-021-01117-y PMC8293573

[53] Shao, M. et al. Inhibition of calcium signaling prevents exhaustion and enhances anti-leukemia efficacy of CAR-T cells via SOCE-Calcineurin-NFAT and glycolysis pathways. Advanced Science **9**, 2103508 (2022). 10.1002/advs.202103508 PMC8948559 35032108

[54] Huang, Y. et al. Inhibition of CD38 enzymatic activity enhances CAR-T cell immune-therapeutic efficacy by repressing glycolytic metabolism. *Cell Rep. Med.* **5**, 101400 (2024). 38307031 10.1016/j.xcrm.2024.101400 PMC10897548

[55] Si, X. et al. Mitochondrial isocitrate dehydrogenase impedes CAR T cell function by restraining antioxidant metabolism and histone acetylation. *Cell Metab.* **36**, 176–192 e110 (2024). 38171332 10.1016/j.cmet.2023.12.010

[56] Weber, E. W. et al. Transient rest restores functionality in exhausted CAR-T cells through epigenetic remodeling. *Science* **372**, eaba1786 (2021). 33795428 10.1126/science.aba1786 PMC8049103

[57] Nair, A. B. & Jacob, S. A simple practice guide for dose conversion between animals and human. *J. Basic Clin. Pharm.* **7**, 27–31 (2016). 27057123 10.4103/0976-0105.177703 PMC4804402

[58] Young, L., Sung, J., Stacey, G. & Masters, J. R. Detection of mycoplasma in cell cultures. *Nat. Protoc.* **5**, 929–934 (2010). 20431538 10.1038/nprot.2010.43

[59] Nicholson, I. C. et al. Construction and characterisation of a functional CD19 specific single chain Fv fragment for immunotherapy of B lineage leukaemia and lymphoma. *Mol. Immunol.* **34**, 1157–1165 (1997). 9566763 10.1016/s0161-5890(97)00144-2

[60] Bolger, A. M., Lohse, M. & Usadel, B. Trimmomatic: a flexible trimmer for Illumina sequence data. *Bioinformatics* **30**, 2114–2120 (2014). 24695404 10.1093/bioinformatics/btu170 PMC4103590

[61] Kim, D., Langmead, B. & Salzberg, S. L. HISAT: a fast spliced aligner with low memory requirements. *Nat. Methods* **12**, 357–360 (2015). 25751142 10.1038/nmeth.3317 PMC4655817

[62] Anders, S., Pyl, P. T. & Huber, W. HTSeq–a Python framework to work with high-throughput sequencing data. *Bioinformatics* **31**, 166–169 (2015). 25260700 10.1093/bioinformatics/btu638 PMC4287950

[63] Robinson, M. D., McCarthy, D. J. & Smyth, G. K. edgeR: a bioconductor package for differential expression analysis of digital gene expression data. *Bioinformatics* **26**, 139–140 (2010). 19910308 10.1093/bioinformatics/btp616 PMC2796818

[64] Lê, S., Josse, J. & Husson, F. FactoMineR: AnRPackage for multivariate analysis. *J. Statis. Software* **25**, 1–18 (2008).

[65] Yu, G., Wang, L. G., Han, Y. & He, Q. Y. clusterProfiler: an R package for comparing biological themes among gene clusters. *OMICS* **16**, 284–287 (2012). 22455463 10.1089/omi.2011.0118 PMC3339379

[66] Huang, D. W., Sherman, B. T. & Lempicki, R. A. Bioinformatics enrichment tools: paths toward the comprehensive functional analysis of large gene lists. *Nucleic Acids Res.* **37**, 1–13 (2009). 19033363 10.1093/nar/gkn923 PMC2615629

[67] Subramanian, A. et al. Gene set enrichment analysis: a knowledge-based approach for interpreting genome-wide expression profiles. *Proc. Natl Acad. Sci. USA* **102**, 15545–15550 (2005). 16199517 10.1073/pnas.0506580102 PMC1239896

[68] Stuart, T. et al. Comprehensive integration of single-cell data. *Cell* **177**, 1888–1902 e1821 (2019). 31178118 10.1016/j.cell.2019.05.031 PMC6687398

[69] Korsunsky, I., Millard, N. & Fan, J. Fast, sensitive and accurate integration of single-cell data with Harmony. *Nat. Methods* **16**, 1289–1296 (2019). 31740819 10.1038/s41592-019-0619-0 PMC6884693

[70] H, W. ggplot2: elegant graphics for data analysis. *Springer-Verlag New York* (2016).

[71] Guo, X. et al. Global characterization of T cells in non-small-cell lung cancer by single-cell sequencing. *Nat. Med.* **24**, 978–985 (2018). 29942094 10.1038/s41591-018-0045-3

[72] Wolf, F. A., Angerer, P. & Theis, F. J. SCANPY: large-scale single-cell gene expression data analysis. *Genome Biol.* **19**, 15 (2018). 29409532 10.1186/s13059-017-1382-0 PMC5802054

[73] Krueger, F. & Andrews, S. R. Bismark: a flexible aligner and methylation caller for Bisulfite-Seq applications. *Bioinformatics* **27**, 1571–1572 (2011). 21493656 10.1093/bioinformatics/btr167 PMC3102221

[74] Zhang, Y. et al. Model-based analysis of ChIP-Seq (MACS). *Genome Biol.* **9**, R137 (2008). 18798982 10.1186/gb-2008-9-9-r137 PMC2592715

[75] Stark, R. & Brown, G. DiffBind: differential binding analysis of ChIP-Seq peak data. *R package version* **100**, 4.3 (2011).

[76] Yu, G., Wang, L. G. & He, Q. Y. ChIPseeker: an R/Bioconductor package for ChIP peak annotation, comparison and visualization. *Bioinformatics* **31**, 2382–2383 (2015). 25765347 10.1093/bioinformatics/btv145

[77] Ramirez, F. et al. deepTools2: a next generation web server for deep-sequencing data analysis. *Nucleic Acids Res.* **44**, W160–W165 (2016). 27079975 10.1093/nar/gkw257 PMC4987876

