# Supplementary material for: MEK inhibition prevents CAR-T cell exhaustion and differentiation via downregulation of c-Fos and JunB
Source: Signal Transduct Target Ther. 2024 Oct 22;9:293. doi: 10.1038/s41392-024-01986-y (PMC11496645; doi:10.1038/s41392-024-01986-y)
Supplement: Supplementary file 1 — Supplementary Figures [file 41392_2024_1986_MOESM1_ESM.pdf]

Supplementary Materials for

MEK inhibition prevents CAR-T cell exhaustion and differentiation via downregulation of c-Fos and JunB

Xiujian Wang[1,2,3,4,7], Xiao Tao[1,2,3,4,7], Pengjie Chen[1,2,3,4,7], Penglei Jiang[2,3,4,6,7], Wenxiao Li[1,2,3,4,7], Hefeng Chang[1,2,3,4], Cong Wei[1,2,3,4], Xinyi Lai[1,2,3,4], Hao Zhang[5], Yihan Pan[1,2,3,4],Lijuan Ding[1,2,3,4], Zuyu Liang[1,2,3,4],Jiazhen Cui[1,2,3,4], Mi Shao[1,2,3,4], Xinyi Teng[1,2,3,4], Tianning Gu[1,2,3,4], Jieping Wei[1,2,3,4], Delin Kong[1,2,3,4], Xiaohui Si[1,2,3,4], Yingli Han[1,2,3,4], Huarui Fu[1,2,3,4], Yu Lin[1,2,3,4], Jian Yu[1,2,3,4], Xia Li[1,2,3,4], Dongrui Wang[1,2,3,4], Yongxian Hu[1,2,3,4], Pengxu Qian[2,3,4,6*], He Huang[1,2,3,4*]

***Corresponding author:**

*He Huang, Professor

E-mail: huanghe@zju.edu.cn

Address:79 Qingchun Road, Hangzhou, Zhejiang Province, China.

Phone number: 86-0571-88208277

*Pengxu Qian, Professor

E-mail: axu@zju.edu.cn

Address:866 Yuhangtang Road, Hangzhou, Zhejiang Province, China.

Phone number: 86-0571-88208195

Supplementary Figures

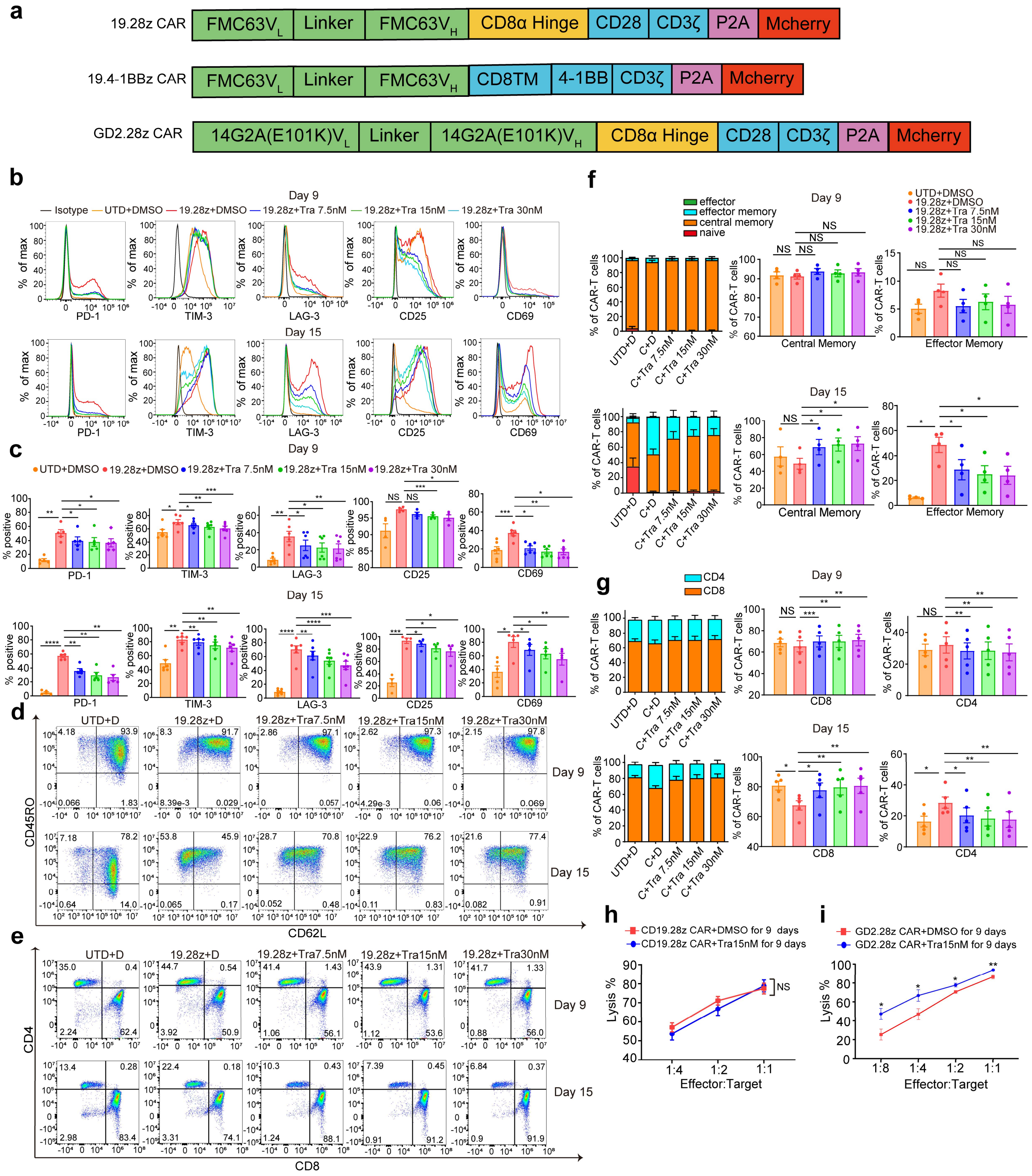

Fig.S1. The dose and time-dependent effects of trametinib on the activation, exhaustion, differentiation, and CD4/CD8 composition of 19.28z CAR-T cells during *ex-vivo* manufacturing.

(**a**) The schematics of CAR constructs in this study. CD19 and GD2 CAR use the single-chain variable fragments from FMC63 and 14G2a antibodies, respectively. Transmembrane (TM) motif and intracellular domains are indicated.

(**b** and **c**) Flow cytometric analysis of exhaustion and activation markers of 19.28z CAR-T cells on day 9 and day 15 of *ex-vivo* culture. The histograms (**b**) of one representative donor are shown. Statistical bar graphs (**c**) are pooled from 4 to 6 donors.

(**d** and **f**) Flow cytometric analysis of the differentiation state of 19.28z CAR-T cells on day 9 and day 15 of *ex-vivo* culture. Pseudocolor plots (**d**) of one representative donor are shown. Bar graphs (**f**) are pooled from 4 donors.

(**e** and **g**) Flow cytometric analysis of the CD8 and CD4 constitution of 19.28z CAR-T cells on day 9 and day 15 of *ex-vivo* culture. Pseudocolor plots (**e**) of one representative donor are shown. Bar graphs (**g**) are pooled from 5 donors.

(**h-i**) Cytotoxicity of 19.28z CAR-T cells (**h**) and GD2.28z CAR-T cells (**i**) pre-treated with trametinib for 9 days. The assay was conducted in culture media without trametinib. Error bars represent means ± SD of triplicate wells. A representative donor from three donors.

Error bars are means ± SEM unless indicated otherwise. Statistical tests were paired one-way ANOVA with Dunnett's multiple comparison test except (**h-i**). For (**h-i**), an unpaired student's t-test was used. *P＜0.05, **P＜0.01,***P＜0.001, ****P＜0.0001, NS: not significant. UTD, Untransduced T. C, 19.28z CAR-T. D, DMSO. Tra, trametinib.

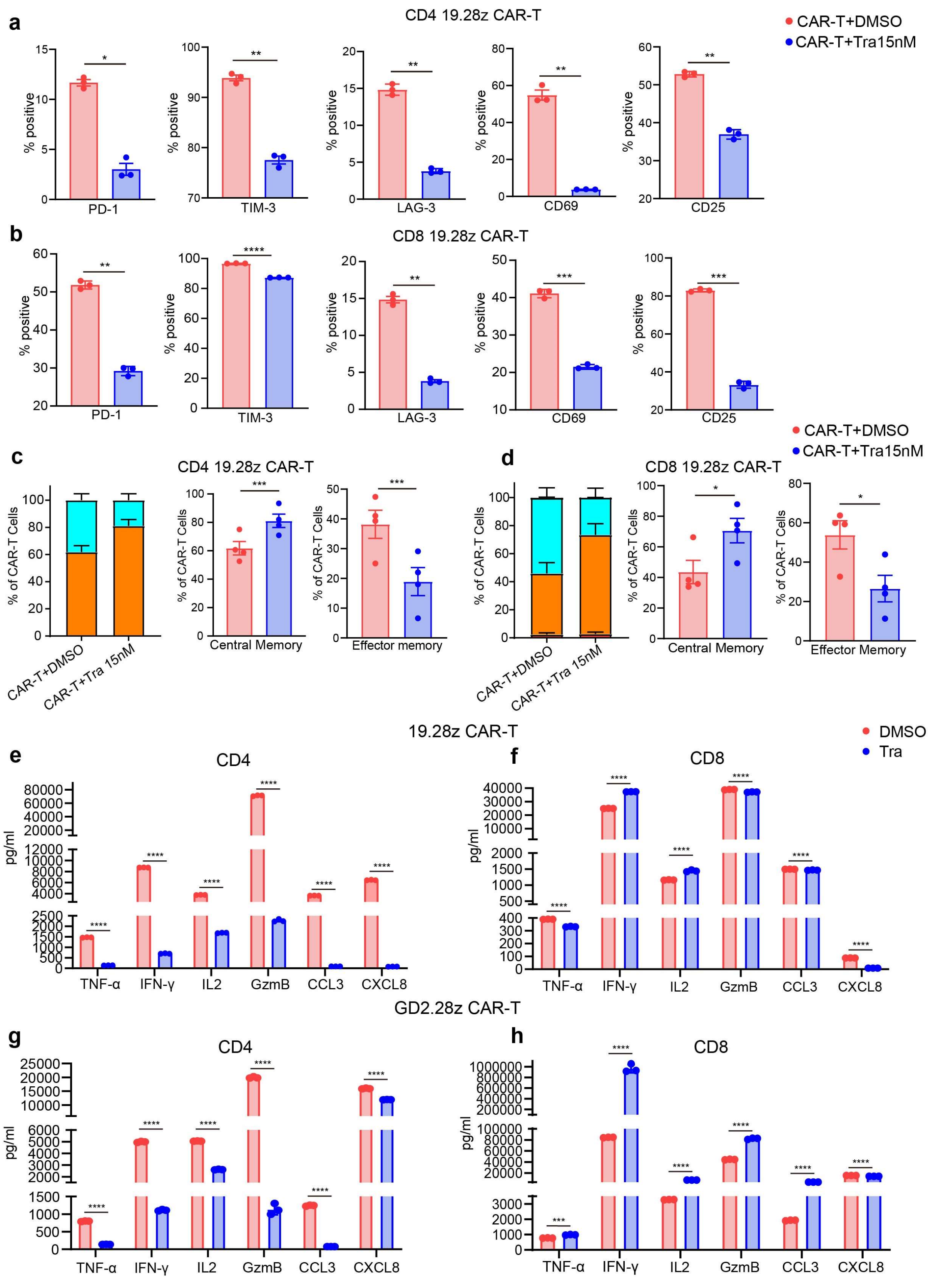
a
CD4 19.28z CAR-T
CAR-T+DMSO
CAR-T+Tra15nM
% positive
PD-1
TIM-3
LAG-3
CD69
CD25
b
CD8 19.28z CAR-T
% positive
PD-1
TIM-3
LAG-3
CD69
CD25
CAR-T+DMSO
CAR-T+Tra15nM
c
CD4 19.28z CAR-T
% of CAR-T Cells
CAR-T+DMSO
CAR-T+Tra 15nM
Central Memory
Effector memory
d
CD8 19.28z CAR-T
% of CAR-T Cells
CAR-T+DMSO
CAR-T+Tra 15nM
Central Memory
Effector Memory
19.28z CAR-T
DMSO
Tra
e
CD4
pg/ml
TNF-α
IFN-γ
IL2
GzmB
CCL3
CXCL8
f
CD8
pg/ml
TNF-α
IFN-γ
IL2
GzmB
CCL3
CXCL8
GD2.28z CAR-T
g
CD4
pg/ml
TNF-α
IFN-γ
IL2
GzmB
CCL3
CXCL8
h
CD8
pg/ml
TNF-α
IFN-γ
IL2
GzmB
CCL3
CXCL8

Fig.S2. The effects of trametinib on CD4 and CD8 CAR-T cells.

(**a-b**) Flow cytometric analysis of exhaustion and activation markers of CD4 19.28z CAR-T cells (**a**) and CD8 19.28z CAR-T cells (**b**) on day 15 of *ex-vivo* culture. Data are pooled from 3 donors.

(**c-d**) Flow cytometric analysis of the differentiation state of CD4 19.28z CAR-T (**c**) cells and CD8 19.28z CAR-T (**d**) cells on day 15 of *ex-vivo* culture. Data are pooled from 4 donors.

(**e-h**) TNF-α, IFN-γ, IL2, granzyme B, CCL3, and CXCL8 secretion of CD4 19.28z (**e**), CD8 19.28z (**f**), CD4 GD2.28z (**g**) and CD8 GD2.28z CAR-T cells (**h**) after 24-h co-culture with tumor cells. Error bars represent means ± SD of triplicate wells. A representative donor from two donors. Error bars are means ± SEM unless indicated otherwise. Statistical tests were paired (**a-d**) or unpaired (**e-h**) student's t-test. $^{*}P<0.05$, $^{**}P<0.01$, $^{***}P<0.001$, $^{****}P<0.0001$.

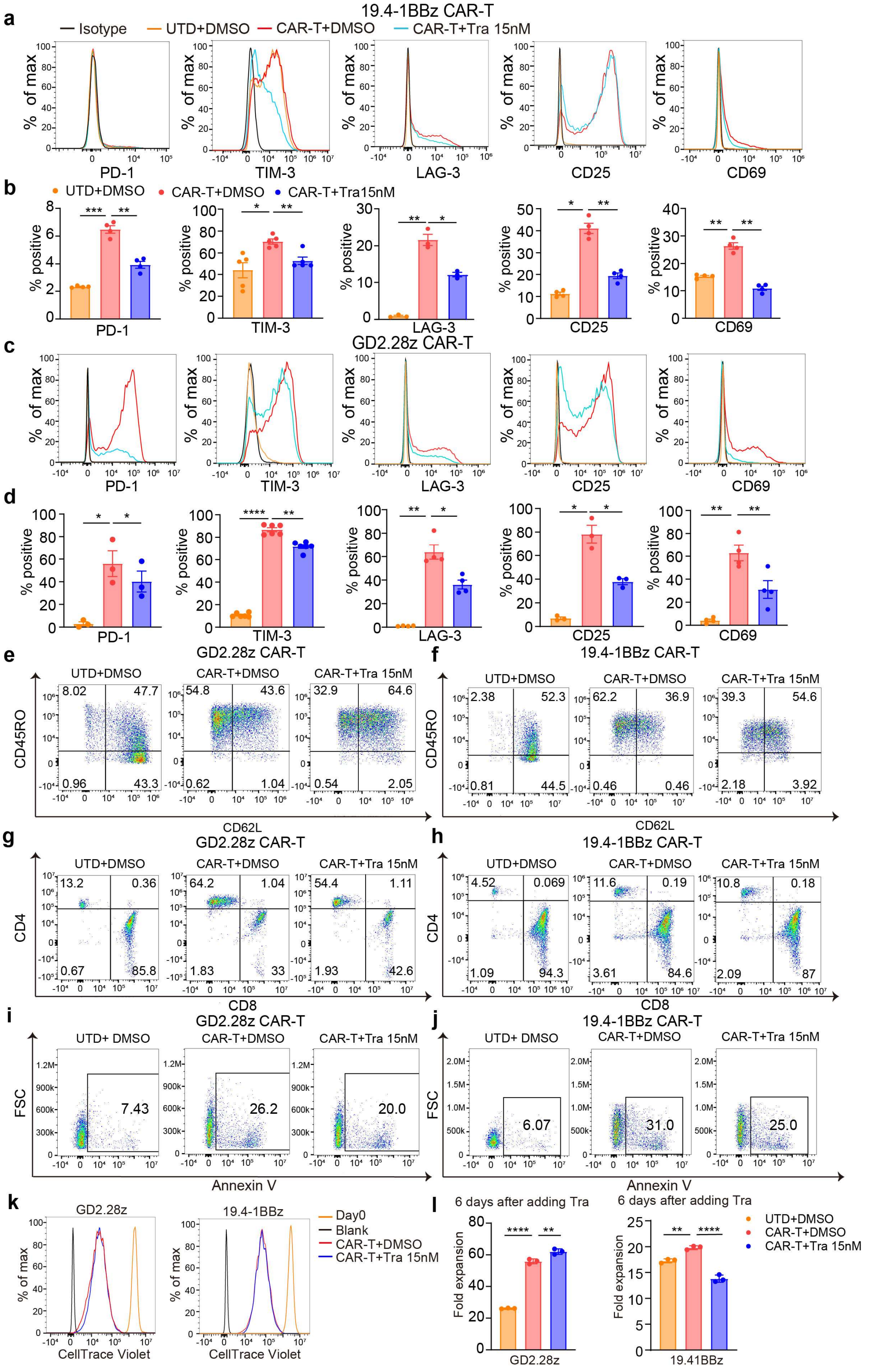

a
19.4-1BBz CAR-T
Isotype
UTD+DMSO
CAR-T+DMSO
CAR-T+Tra 15nM
% of max
PD-1
TIM-3
LAG-3
CD25
CD69
b
UTD+DMSO
CAR-T+DMSO
CAR-T+Tra15nM
% positive
c
GD2.28z CAR-T
d
e
GD2.28z CAR-T
UTD+DMSO
CAR-T+DMSO
CAR-T+Tra 15nM
CD45RO
CD62L
8.02
47.7
0.96
43.3
54.8
43.6
0.62
1.04
32.9
64.6
0.54
2.05
f
19.4-1BBz CAR-T
2.38
52.3
0.81
44.5
62.2
36.9
0.46
0.46
39.3
54.6
2.18
3.92
g
GD2.28z CAR-T
CD4
CD8
13.2
0.36
0.67
85.8
64.2
1.04
1.83
33
54.4
1.11
1.93
42.6
h
19.4-1BBz CAR-T
4.52
0.069
1.09
94.3
11.6
0.19
3.61
84.6
10.8
0.18
2.09
87
i
GD2.28z CAR-T
UTD+ DMSO
FSC
Annexin V
7.43
26.2
20.0
j
19.4-1BBz CAR-T
6.07
31.0
25.0
k
GD2.28z
19.4-1BBz
Day0
Blank
CAR-T+DMSO
CAR-T+Tra 15nM
% of max
CellTrace Violet
l
6 days after adding Tra
Fold expansion
GD2.28z
19.41BBz
UTD+DMSO
CAR-T+DMSO
CAR-T+Tra 15nM

Fig.S3. Trametinib restrains the activation, exhaustion, apoptosis, CD8 decrease, and differentiation of GD2.28z CAR-T cells and 19.4-1BBz CAR-T cells induced by tonic signaling.

(**a-d**) Flow cytometric analysis of exhaustion and activation markers of 19.4-1BBz CAR-T cells and GD2.28z CAR-T cells on day 15 of *ex-vivo* culture. The histograms (**a** and **c**) of one representative donor are shown. Error bars in (**b**) and (**d**) are means ± SEM; data are pooled from 3 to 6 donors.

(**e-j**) The differentiation state (**e** and **f**), CD8 and CD4 composition (**g** and **h**), and Annexin V positive rate (**i** and **j**) of GD2.28z CAR-T cells and 19.4-1BBz CAR-T cells on day 15 of *ex-vivo* culture. Pseudocolor plots of one representative donor are shown (n = 3 donors).

(**k**) Representative histogram of CellTrace Violet showing the cell proliferation state of GD2.28z and 19.4-1BBz CAR-T cells after a six-day treatment with trametinib. N=2 donors.

(**l**) The bar graphs show the expansion fold of GD2.28z CAR-T cells and 19.4-1BBz CAR-T cells. Data are means ± SD of triplicate wells from one representative donor (n = 3 donors).

The statistical tests were paired (**b** and **d**) or unpaired (**l**) two-tailed one-way ANOVA with Dunnett's multiple comparison test. *P＜0.05, **P＜0.01,***P＜0.001, ****P＜0.0001. UTD, Untransduced T. Tra, trametinib.

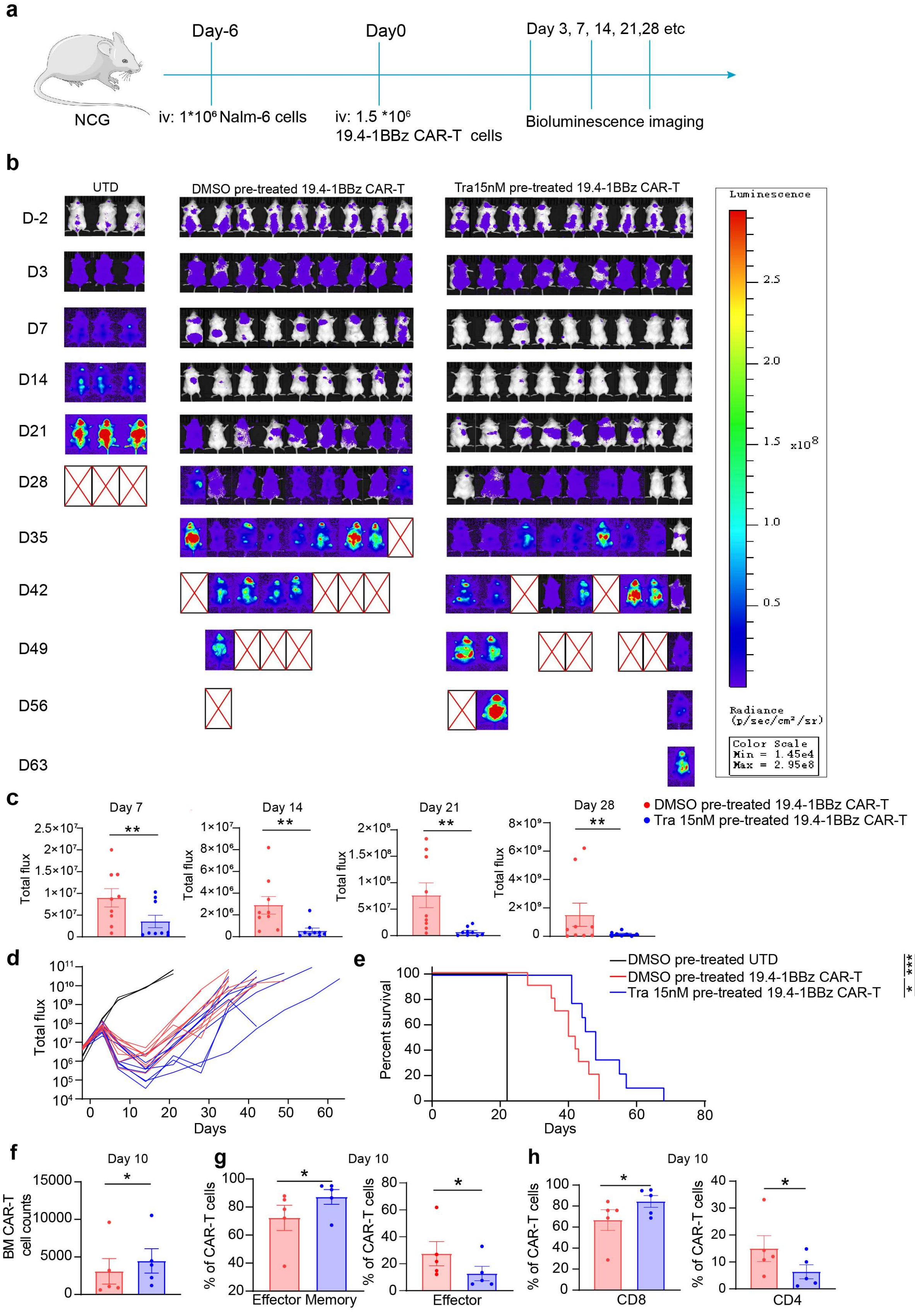
a
NCG
Day-6
Day0
Day 3, 7, 14, 21,28 etc
iv: 1*10⁶ Nalm-6 cells
iv: 1.5 *10⁶ 19.4-1BBz CAR-T cells
Bioluminescence imaging
b
UTD
DMSO pre-treated 19.4-1BBz CAR-T
Tra15nM pre-treated 19.4-1BBz CAR-T
D-2
D3
D7
D14
D21
D28
D35
D42
D49
D56
D63
Luminescence
x10⁸
Radiance (p/sec/cm²/sr)
Color Scale Min = 1.45e4 Max = 2.95e8
c
Day 7
Day 14
Day 21
Day 28
Total flux
DMSO pre-treated 19.4-1BBz CAR-T
Tra 15nM pre-treated 19.4-1BBz CAR-T
d
Total flux
Days
e
DMSO pre-treated UTD
DMSO pre-treated 19.4-1BBz CAR-T
Tra 15nM pre-treated 19.4-1BBz CAR-T
Percent survival
Days
f
Day 10
BM CAR-T cell counts
g
Day 10
% of CAR-T cells
Effector Memory
Effector
h
Day 10
CD8
CD4

Fig.S4. Trametinib pretreatment during *ex-vivo* manufacturing enhances the *in-vivo* efficacy of CD19.4-1BBz CAR-T cells.

(**a**) 1.5×10^6 CD19.4-1BBz CAR-T cells manufactured in the presence of trametinib 15nM or DMSO for 9 days were infused intravenously (IV) to NCG mice 6 days after engraftment of 1×10^6 Nalm-6-GL leukemia cells.

(**b-d**) Tumor growth was monitored by bioluminescent imaging (UTD cells: n=3; DMSO-treated CD19.4-1BBz CAR-T cells: n=9; trametinib 15nM-treated CD19.4-1BBz CAR-T cells: n=9). Each dot in (**c**) and each curve in (**d**) represents one mouse. D, day.

(**e**) Kaplan–Meier analysis of survival of mice from (**b**).

(**f-h**) The number (**f**), T_{EM} and T_E percentage (**g**), and CD8/CD4 composition (**h**) of CAR-T cells in the bone marrow 10 days after CAR-T infusion. Data are from 1 representative experiment. Each dot represents one mouse. N=5.

Error bars are means ± SEM. Statistical analysis was a two-tailed Wilcoxon test (**c**) and paired two-tailed student's t-test (**f-h**). Survival curves were compared using a log-rank Mantel-Cox test. *P＜0.05, **P＜0.01. D, day. Tra, trametinib. UTD, untransduced T.

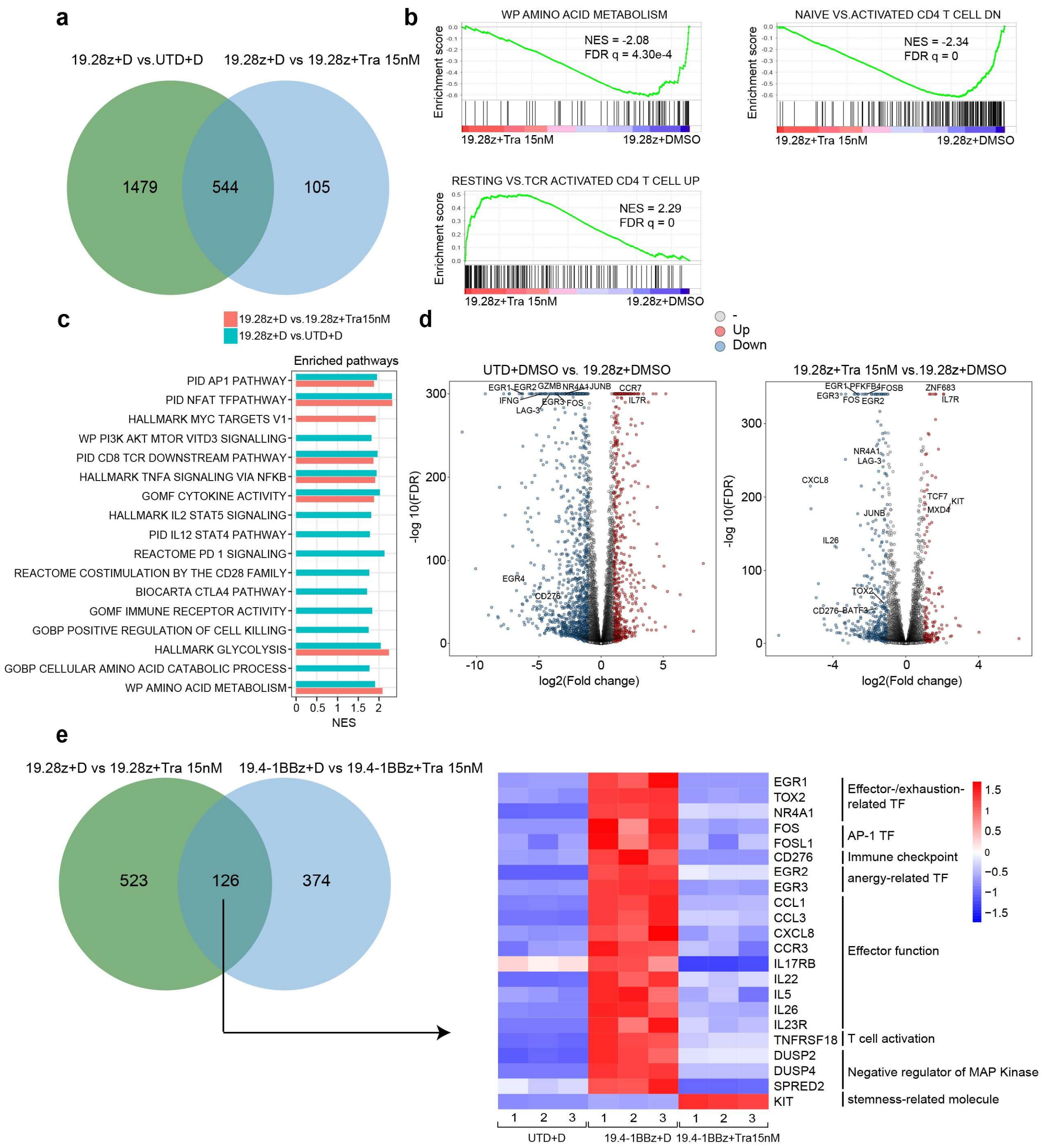

Fig.S5. MEK inhibition modifies the gene-expression profile of CAR-T cells during *ex-vivo* manufacturing.

(**a**) Venn diagram displaying overlap of differentially expressed genes (DEGs) between 19.28z CAR-T+DMSO versus UTD+DMSO and 19.28z CAR-T+DMSO versus 19.28z CAR-T+trametinib 15nM.

(**b**) Representative GSEA enrichment plot.

(**c**) Normalized enrichment score (NES) of significantly upregulated gene sets in DMSO-treated 19.28z CAR-T cells relative to UTD cells and trametinib-treated 19.28z CAR-T cells as determined by GSEA using the MSigDB H, C2, and C5 gene sets. For all pathways, the false discovery rate (FDR) <0.05.

(**d**) The volcano plots illustrate DEGs in UTD+DMSO versus 19.28z+DMSO and 19.28z +Tra15nM versus 19.28z+DMSO. Red and blue dots indicate significant genes with FDR<0.05 and fold change >2.

(**e**) Venn diagram and heatmap showing overlap of differentially expressed genes (DEGs) between 19.28z CAR-T+DMSO versus 19.28z CAR-T+trametinib 15nM and 19.4-1BBz CAR-T+DMSO versus 19.4-1BBz CAR-T+trametinib 15nM.

For all analyses, n = 3 per group. UTD, Untransduced T. D, DMSO. Tra, trametinib.

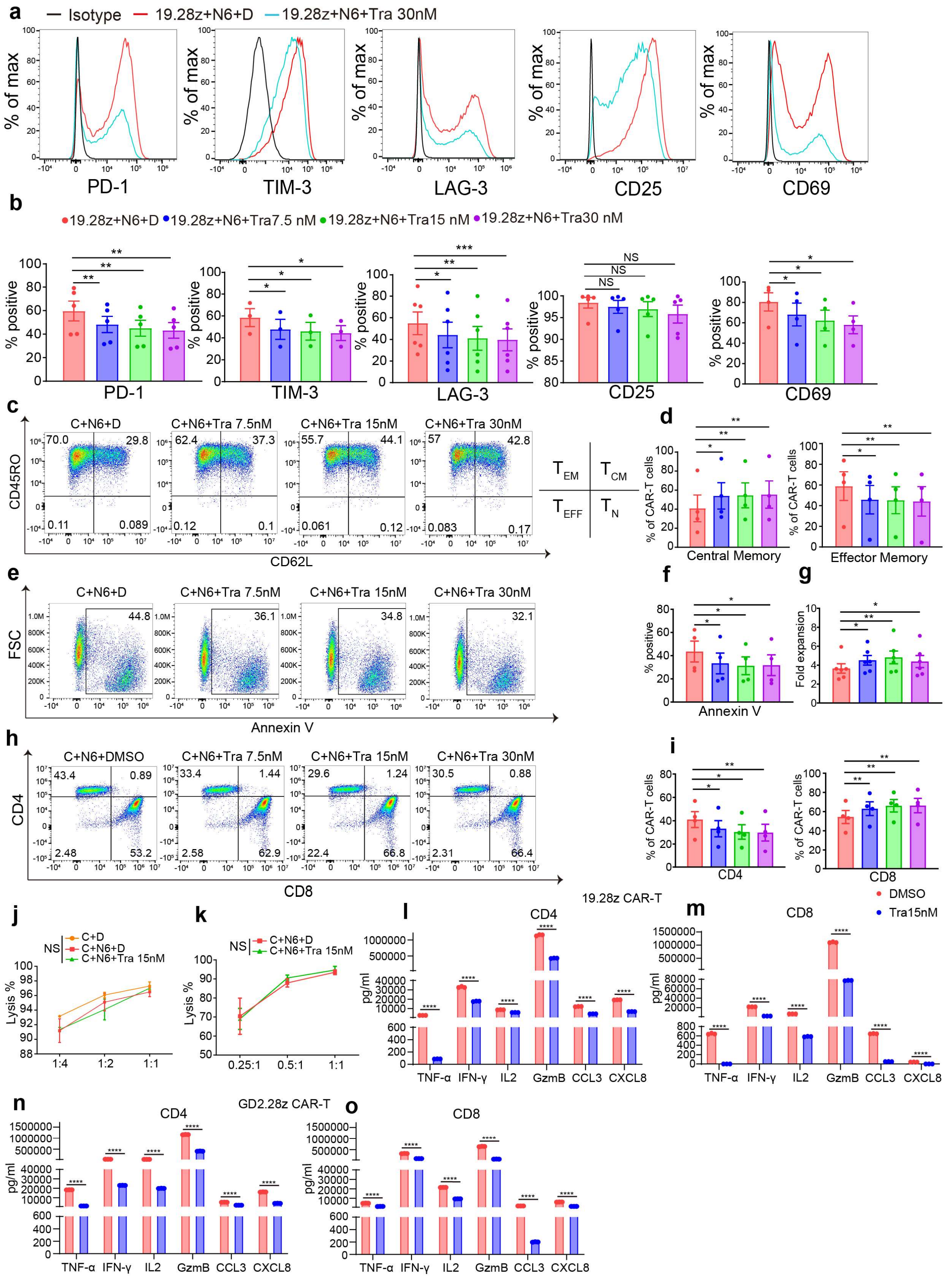
a
Isotype
19.28z+N6+D
19.28z+N6+Tra 30nM
% of max
PD-1
TIM-3
LAG-3
CD25
CD69
b
19.28z+N6+D
19.28z+N6+Tra7.5 nM
19.28z+N6+Tra15 nM
19.28z+N6+Tra30 nM
% positive
NS
c
C+N6+D
C+N6+Tra 7.5nM
C+N6+Tra 15nM
C+N6+Tra 30nM
CD45RO
CD62L
70.0
29.8
0.11
0.089
62.4
37.3
0.12
0.1
55.7
44.1
0.061
0.12
57
42.8
0.083
0.17
T_EM
T_CM
T_EFF
T_N
d
% of CAR-T cells
Central Memory
Effector Memory
e
FSC
Annexin V
44.8
36.1
34.8
32.1
f
g
Fold expansion
h
C+N6+DMSO
CD4
CD8
43.4
0.89
2.48
53.2
33.4
1.44
2.58
62.9
29.6
1.24
22.4
66.8
30.5
0.88
2.31
66.4
i
j
C+D
C+N6+D
C+N6+Tra 15nM
Lysis %
k
l
19.28z CAR-T
m
DMSO
Tra15nM
pg/ml
TNF-α
IFN-γ
IL2
GzmB
CCL3
CXCL8
n
GD2.28z CAR-T
o

Fig.S6. Trametinib limits the exhaustion and differentiation of CAR-T cells triggered by target antigen.

(**a**-**b**) Flow cytometric analysis of the exhaustion and activation markers of 19.28z CAR-T cells. The histograms (**a**) of one representative donor are shown. Bar graphs (**b**) are pooled from 3 to 6 donors.

(**c-d**) The differentiation state of 19.28z CAR-T cells. Pseudocolor plots (**c**) of one representative donor are shown. Bar graphs (**d**) are pooled from 4 donors.

(**e**-**f**) Quantification of apoptosis of 19.28z CAR T cells. One representative donor's pseudocolor plots (**e**) are shown, and the bar graph (**f**) is pooled from 4 donors.

(**g**) The bar graphs show the expansion fold of 19.28z CAR-T cells. Data are pooled from 6 donors.

(**h**-**i**) CD8 and CD4 composition in 19.28z CAR-T cells. One representative donor's pseudocolor plots (**h**) are shown, and the bar graphs (**i**) are pooled from 4 independent donors.

(**j**-**k**) Cytotoxicity of 19.28z CAR-T cells co-cultured with Nalm-6 GL cells for 18 hours in the culture media free of trametinib or DMSO. The CAR-T cells used for the assay were treated by the protocol indicated in the upper (**j**) and middle (**k**) panels of Fig.4**a**. Error bars represent means ± SD of triplicate wells. Representative donor from three (**j**) or two donors (**k**).

(**l-o**) TNF-α, IFN-γ, IL2, granzyme B, CCL3, and CXCL8 release of CD4 19.28z (**l**), CD8 19.28z (**m**), CD4 GD2.28z (**n**) and CD8 GD2.28z (**o**) CAR-T cells after 24-h co-culture with tumor cells. Error bars represent means ± SD of triplicate wells. Representative donor from three (**l-m**) or two (**n-o**) donors.

The data in (**a-i**) were produced using CAR-T cells treated by protocols indicated in the middle panel of Fig.4**a**. Error bars are mean ± SEM unless indicated otherwise. Statistical analysis was paired (**b, d, f, g** and **i**) or unpaired (**j**) one-way ANOVA with Dunnett's multiple comparison test

and unpaired students’ t-test (**k-o**) *P＜0.05, **P＜0.01, ***P＜0.001, ****P＜0.0001, NS: not significant. C, 19.28z CAR-T. D, DMSO. Tra, trametinib. N6, Nalm-6.

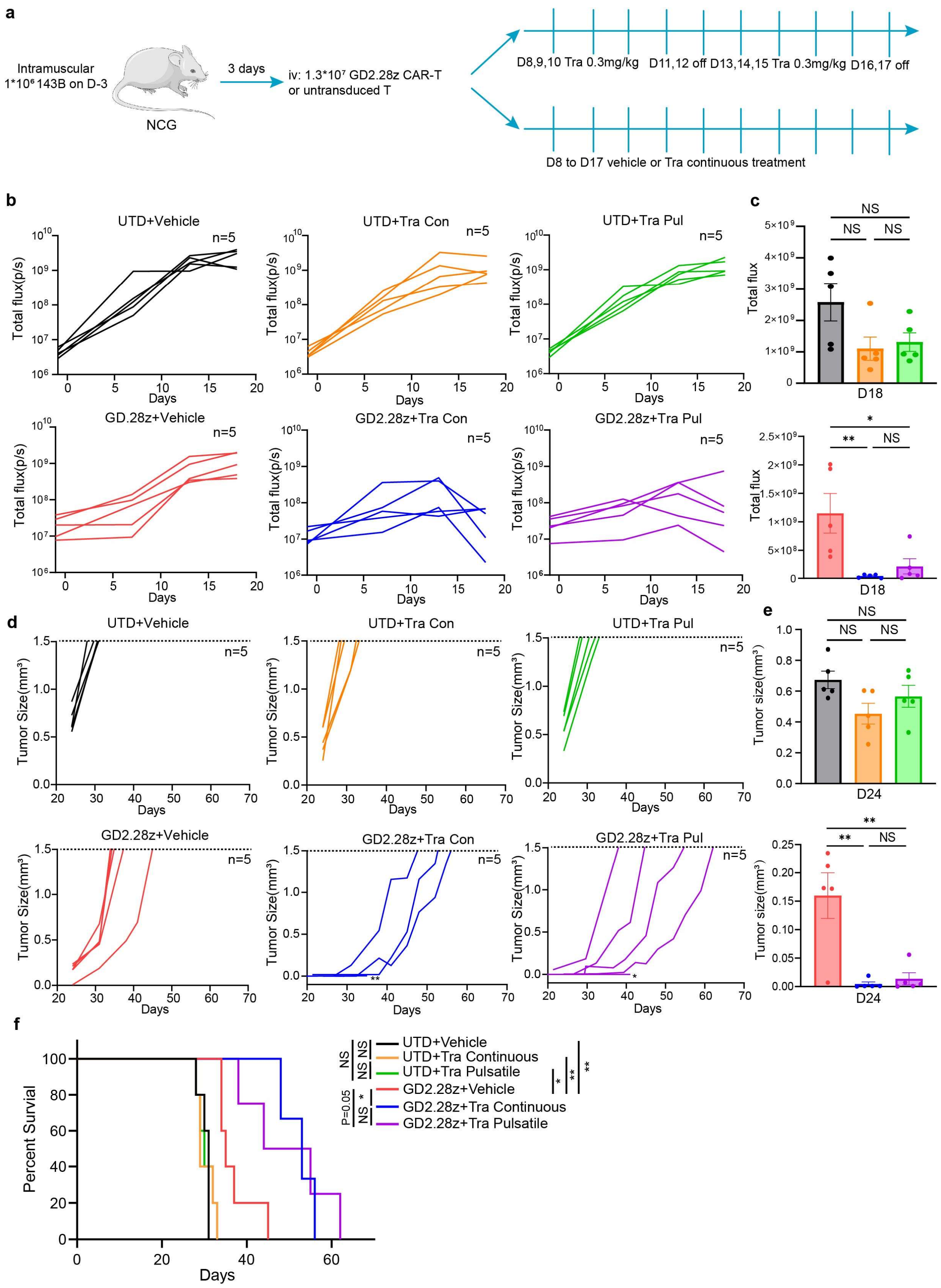

a
Intramuscular
1*10⁶ 143B on D-3
NCG
3 days
iv: 1.3*10⁷ GD2.28z CAR-T
or untransduced T
D8,9,10 Tra 0.3mg/kg D11,12 off D13,14,15 Tra 0.3mg/kg D16,17 off
D8 to D17 vehicle or Tra continuous treatment
b
UTD+Vehicle
UTD+Tra Con
UTD+Tra Pul
GD.28z+Vehicle
GD2.28z+Tra Con
GD2.28z+Tra Pul
n=5
Total flux(p/s)
Days
c
Total flux
D18
NS
*
**
d
Tumor Size(mm³)
Days
UTD+Vehicle
UTD+Tra Con
UTD+Tra Pul
GD2.28z+Vehicle
GD2.28z+Tra Con
GD2.28z+Tra Pul
n=5
e
Tumor size(mm³)
D24
NS
**
f
Percent Survial
Days
UTD+Vehicle
UTD+Tra Continuous
UTD+Tra Pulsatile
GD2.28z+Vehicle
GD2.28z+Tra Continuous
GD2.28z+Tra Pulsatile
P=0.05
NS
*
**

Fig.S7. In-vivo administration of trametinib enhances the antitumor activities of CAR-T cells by offsetting CAR-T cell exhaustion and differentiation

(**a**) Mice treated with GD2.28z CAR-T cells or UTD cells received either continuous vehicle, continuous trametinib, or pulsatile trametinib.

(**b-c**) Tumor growth was monitored by bioluminescent imaging. Each curve in (**b**) and each dot in (**c**) represents one mouse. N=5 for each group. D, day.

(**d**) Growth curve of tumor size in mice from (**b**). Each line represents one mouse. The dotted lines indicate the endpoint where tumor volume reached $1.5cm^3$, and the asterisks indicate mice died before reaching the endpoint. In the GD2.28z + Tra continuous group, two mice died accidentally; in the GD2.28z + Tra pulsatile group, one mouse died accidentally.

(**e**) Bar plots of tumor volume of mice from (**d**). N=5 mice per group.

(**f**) Kaplan–Meier analysis of survival of mice from (**b**). The mice that died accidentally before reaching the endpoint were excluded from the Kaplan-Meier analysis.

Error bars are means ± SEM. Statistical analysis was paired one-way ANOVA with Dunnett's multiple comparison test (**c** and **e**). Survival curves were compared using a log-rank Mantel-Cox test (**f**). *P＜0.05, **P＜0.01. NS: not significant. D, day. Tra, trametinib. UTD, untransduced T. Con, continuous. Pul, pulsatile.

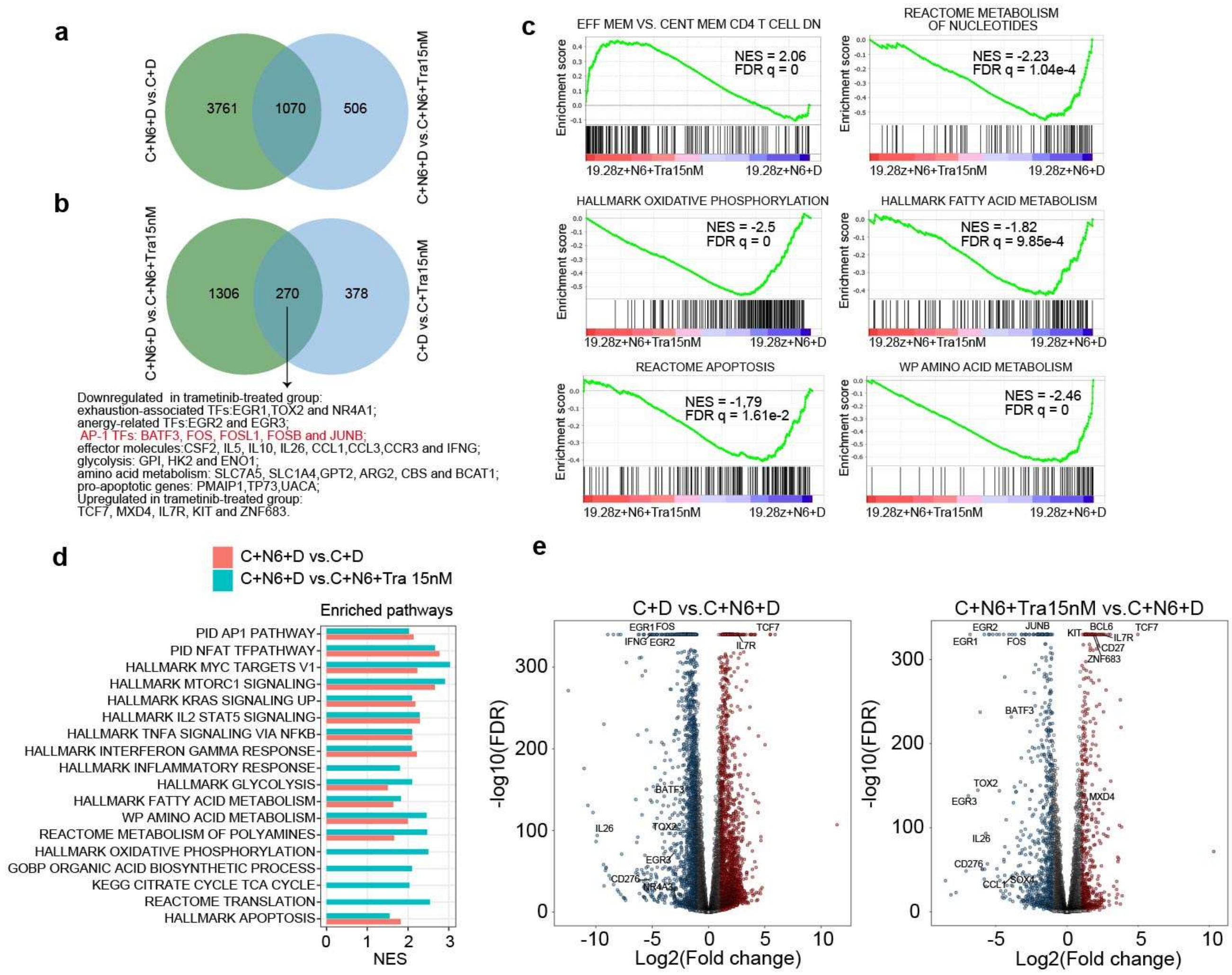

Fig.S8. MEK inhibition reprograms the gene profile of antigen-stimulated CAR-T cells into a naïve-/memory-like state.

(**a**) Venn diagram displaying overlap of DEGs between 19.28z CAR-T+Nalm-6+DMSO versus 19.28z CAR-T+DMSO and 19.28z CAR-T+Nalm-6+DMSO versus 19.28z CAR-T+Nalm-6+trametinib 15nM.

(**b**) Venn diagram displaying overlap of DEGs between 19.28z CAR-T+Nalm-6+DMSO versus 19.28z CAR-T+Nalm-6+trametinib 15nM and 19.28z CAR-T+DMSO versus 19.28z CAR-T+trametinib 15nM. The AP-1 TFs are denoted in red.

(**c**) Representative GSEA enrichment plot.

(**d**) NES of significantly upregulated gene sets in 19.28z CAR-T+Nalm-6+DMSO relative to 19.28z CAR-T+DMSO and 19.28z CAR-T+Nalm-6+trametinib 15nM as determined by GSEA using the MSigDB H, C2, and C5 gene sets. For all pathways, the FDR <0.05.

(**e**) The volcano plots illustrate DEGs in 19.28z CAR-T+DMSO versus 19.28z CAR-T+Nalm-6+DMSO and 19.28z CAR-T+Nalm-6+trametinib 15nM versus 19.28z CAR-T+Nalm-6+DMSO. Red and blue dots indicate significant genes with FDR<0.05 and fold change >2.

For all analyses, n = 3 per group. C, 19.28z CAR-T. N6, Nalm-6. D, DMSO. Tra, trametinib.

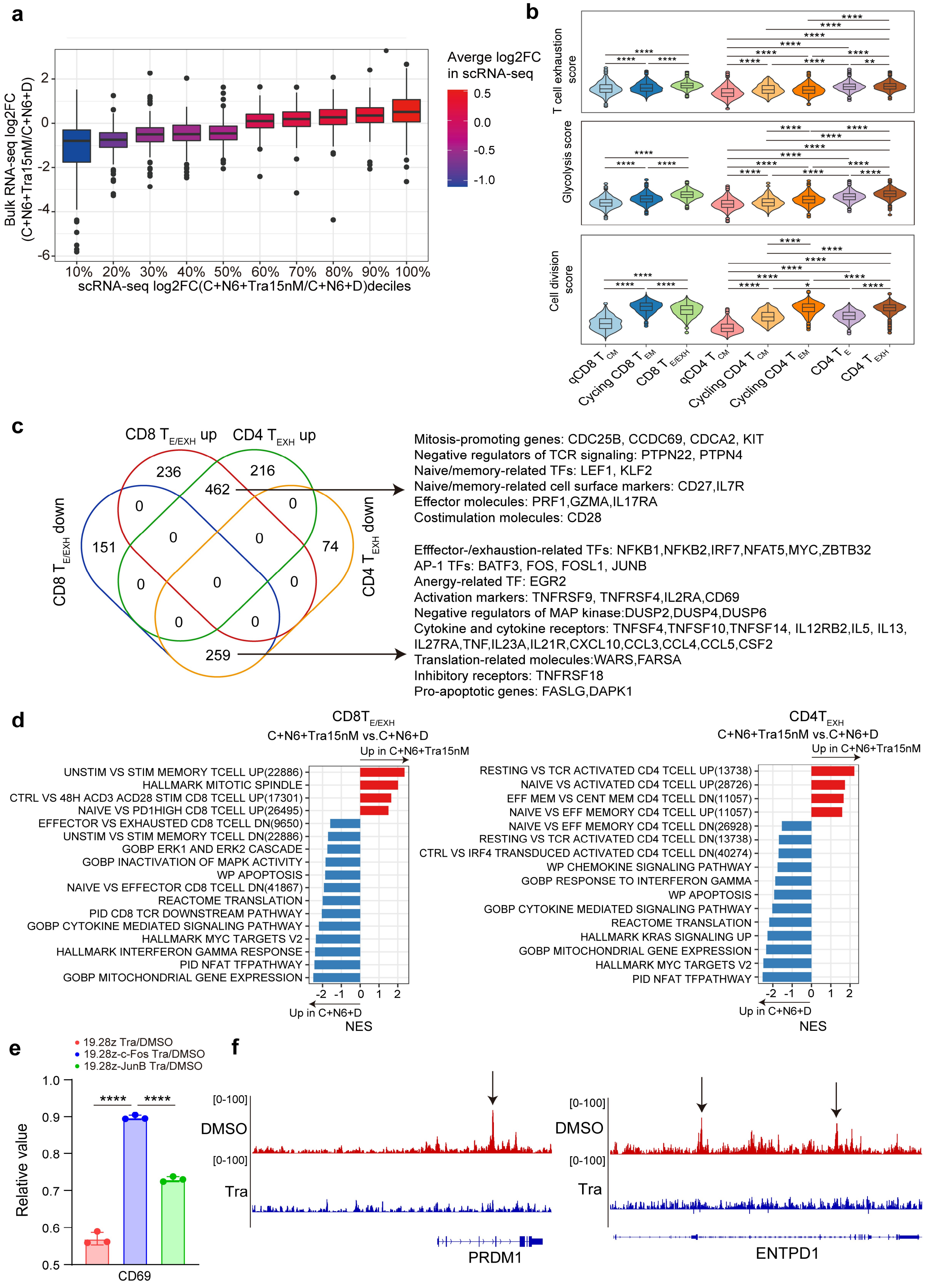
a
Averge log2FC
in scRNA-seq
0.5
0.0
-0.5
-1.0
Bulk RNA-seq log2FC
(C+N6+Tra15nM/C+N6+D)
10% 20% 30% 40% 50% 60% 70% 80% 90% 100%
scRNA-seq log2FC(C+N6+Tra15nM/C+N6+D)deciles
b
T cell exhaustion score
Glycolysis score
Cell division score
qCD8 T CM
Cycling CD8 T EM
CD8 T E/EXH
qCD4 T CM
Cycling CD4 T CM
Cycling CD4 T EM
CD4 T E
CD4 T EXH
c
CD8 T E/EXH up
CD4 T EXH up
CD8 T E/EXH down
CD4 T EXH down
236
216
462
151
74
259
Mitosis-promoting genes: CDC25B, CCDC69, CDCA2, KIT
Negative regulators of TCR signaling: PTPN22, PTPN4
Naive/memory-related TFs: LEF1, KLF2
Naive/memory-related cell surface markers: CD27,IL7R
Effector molecules: PRF1,GZMA,IL17RA
Costimulation molecules: CD28
Efffector-/exhaustion-related TFs: NFKB1,NFKB2,IRF7,NFAT5,MYC,ZBTB32
AP-1 TFs: BATF3, FOS, FOSL1, JUNB
Anergy-related TF: EGR2
Activation markers: TNFRSF9, TNFRSF4,IL2RA,CD69
Negative regulators of MAP kinase:DUSP2,DUSP4,DUSP6
Cytokine and cytokine receptors: TNFSF4,TNFSF10,TNFSF14, IL12RB2,IL5, IL13,
IL27RA,TNF,IL23A,IL21R,CXCL10,CCL3,CCL4,CCL5,CSF2
Translation-related molecules:WARS,FARSA
Inhibitory receptors: TNFRSF18
Pro-apoptotic genes: FASLG,DAPK1
d
CD8T E/EXH
C+N6+Tra15nM vs.C+N6+D
Up in C+N6+Tra15nM
UNSTIM VS STIM MEMORY TCELL UP(22886)
HALLMARK MITOTIC SPINDLE
CTRL VS 48H ACD3 ACD28 STIM CD8 TCELL UP(17301)
NAIVE VS PD1HIGH CD8 TCELL UP(26495)
EFFECTOR VS EXHAUSTED CD8 TCELL DN(9650)
UNSTIM VS STIM MEMORY TCELL DN(22886)
GOBP ERK1 AND ERK2 CASCADE
GOBP INACTIVATION OF MAPK ACTIVITY
WP APOPTOSIS
NAIVE VS EFFECTOR CD8 TCELL DN(41867)
REACTOME TRANSLATION
PID CD8 TCR DOWNSTREAM PATHWAY
GOBP CYTOKINE MEDIATED SIGNALING PATHWAY
HALLMARK MYC TARGETS V2
HALLMARK INTERFERON GAMMA RESPONSE
PID NFAT TFPATHWAY
GOBP MITOCHONDRIAL GENE EXPRESSION
Up in C+N6+D
NES
CD4T EXH
C+N6+Tra15nM vs.C+N6+D
Up in C+N6+Tra15nM
RESTING VS TCR ACTIVATED CD4 TCELL UP(13738)
NAIVE VS ACTIVATED CD4 TCELL UP(28726)
EFF MEM VS CENT MEM CD4 TCELL DN(11057)
NAIVE VS EFF MEMORY CD4 TCELL UP(11057)
NAIVE VS EFF MEMORY CD4 TCELL DN(26928)
RESTING VS TCR ACTIVATED CD4 TCELL DN(13738)
CTRL VS IRF4 TRANSDUCED ACTIVATED CD4 TCELL DN(40274)
WP CHEMOKINE SIGNALING PATHWAY
GOBP RESPONSE TO INTERFERON GAMMA
WP APOPTOSIS
GOBP CYTOKINE MEDIATED SIGNALING PATHWAY
REACTOME TRANSLATION
HALLMARK KRAS SIGNALING UP
GOBP MITOCHONDRIAL GENE EXPRESSION
HALLMARK MYC TARGETS V2
PID NFAT TFPATHWAY
Up in C+N6+D
NES
e
19.28z Tra/DMSO
19.28z-c-Fos Tra/DMSO
19.28z-JunB Tra/DMSO
Relative value
CD69
f
[0-100]
DMSO
[0-100]
Tra
PRDM1
ENTPD1

Fig.S9. Single-cell transcriptome and Cut and Tag analysis.

(**a**) Decile plot showed the correlation between bulk RNA-seq and single-cell RNA-seq datasets. Genes differentially expressed in single-cell data between trametinib- and DMSO-treated groups were divided into 10 groups and sorted by single-cell fold-change, going from the lowest to the highest (x-axis). Y-axis plots the fold change (trametinib/DMSO) in the bulk RNA-seq for genes in each group.

(**b**) The cell division, glycolysis, and T cell exhaustion score among the eight clusters.

The statistical test was a two-tailed Wilcoxon test. *P＜0.05, **P＜0.01, ****P＜0.0001.

(**c**) Venn diagram displayed overlap of DEGs in CD4 T_{EXH} and CD8 $T_{E/EXH}$ comparing trametinib versus DMSO.

(**d**) NES of significantly up- or downregulated gene sets in CD8 $T_{E/EXH}$ (left panel) and CD4 T_{EXH} (right panel) in the comparison of 19.28z CAR-T+Nalm-6+trametinib 15nM versus 19.28z CAR-T+Nalm-6+DMSO, as determined by GSEA using the MSigDB H, C2, C5, and C7 gene sets. For all pathways, the FDR <0.05.

(**e**) Flow cytometric analysis of CD69 of CAR-T cells (n=3). Error bars are mean ± SEM. The statistical test was an ordinary one-way ANOVA with Dunnett's multiple comparison test.****P ＜0.0001. Data are normalized to DMSO-treated group.

(**f**) Representative Cut & Tag sequencing tracks showing binding of JunB to PRDM1 (encoding BLIMP-1) and ENTPD1 (encoding CD39) in DMSO- and trametinib-treated CAR-T cells. Arrows denote significant peaks.

C, 19.28z CAR-T. N6, Nalm-6. D, DMSO. Tra, trametinib.

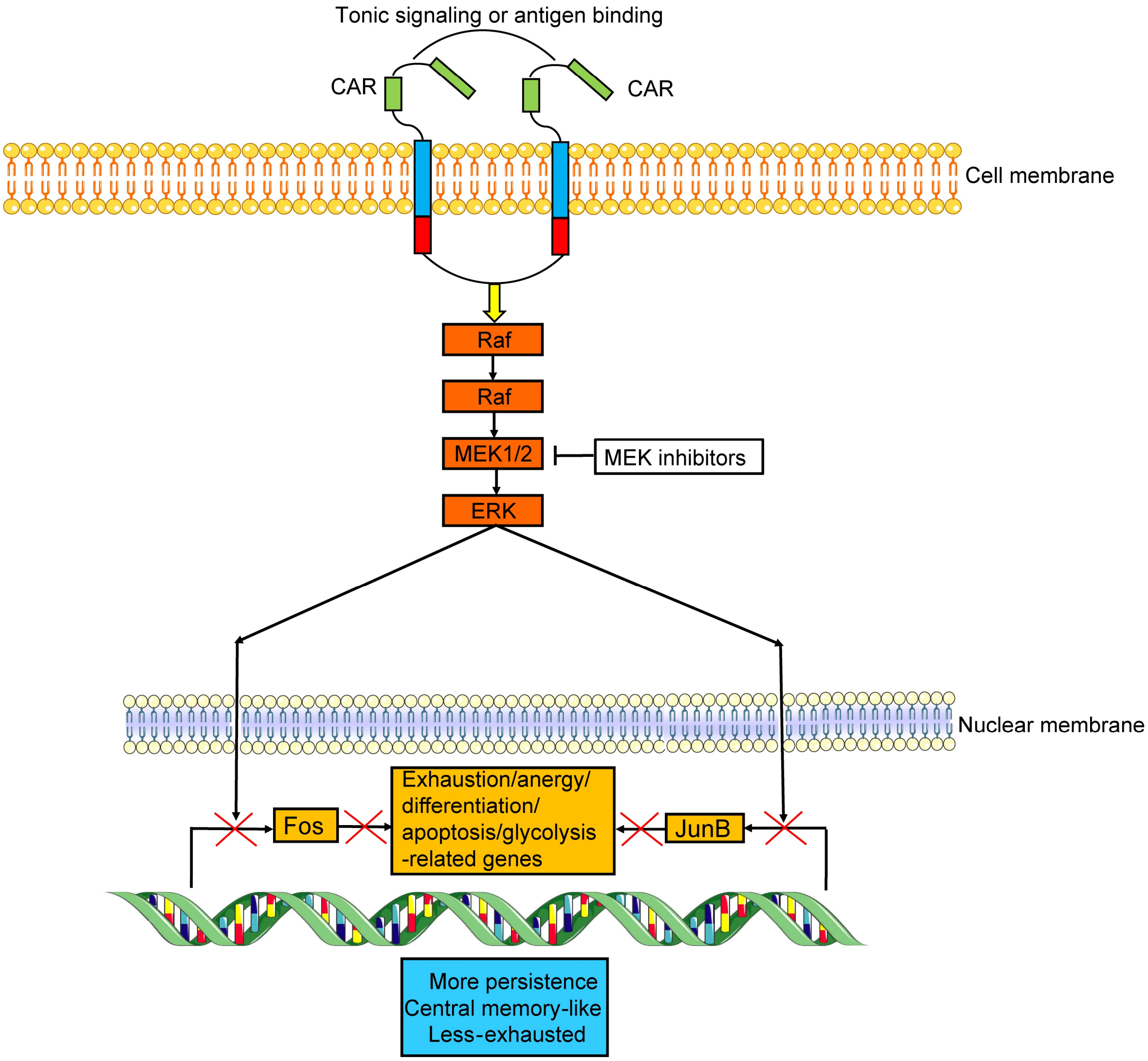

Fig.S10. Proposed mechanism of MEKI in offsetting CAR-T cell exhaustion and differentiation triggered by tonic signaling and antigen binding.
