# Supplementary material for: MEK inhibition prevents CAR-T cell exhaustion and differentiation via downregulation of c-Fos and JunB
Source: Signal Transduct Target Ther. 2024 Oct 22;9:293. doi: 10.1038/s41392-024-01986-y (PMC11496645; doi:10.1038/s41392-024-01986-y)
Supplement: Supplementary file 4 — Supplementary table legend [file 41392_2024_1986_MOESM4_ESM.docx]

**Supplementary Table 1.** Differentially expressed genes among DMSO-treated UTD cells, DMSO-treated 19.28z CAR-T cells, and trametinib-treated 19.28z CAR-T cells from bulk RNA-seq experiments.

**Supplementary Table 2.** Differentially expressed genes among resting 19.28z CAR-T cells and Nalm-6-stimulated 19.28z CAR-T cells treated with DMSO or trametinib from bulk RNA-seq experiments.
